# Metal Nanoparticle-Reinforced Hydrogels Applied in the Inhibition of Clinical Pathogens: Structural Features, Mechanisms, and Biomedical Prospects

**DOI:** 10.3390/pharmaceutics18060765

**Published:** 2026-06-22

**Authors:** Lizeth Geraldine Muñoz, Yhors Ciro, Andrés Felipe Chamorro

**Affiliations:** 1 Grupo de Investigación en Electroquímica y Medio Ambiente (GIEMA), Facultad de Ciencias Básicas, Universidad Santiago de Cali, Cali 760035, Colombia; lizeth.munoz02@usc.edu.co; 2 Grupo de Investigación en Química y Biotecnología (QUIBIO), Facultad de Ciencias Básicas, Universidad Santiago de Cali, Cali 760035, Colombia; 3 Grupo de Investigación en Química Ambiental y Tecnologías Limpias (QUATELI), Departamento de Química, Facultad de Ciencias Básicas y Aplicadas, Universidad Militar Nueva Granada, Cajicá 250247, Colombia

**Keywords:** hydrogels, metallic nanoparticles, antimicrobial resistance, wound healing, green synthesis, biomedical nanomaterials, drug delivery

## Abstract

The increasing prevalence of antimicrobial resistance (AMR) has promoted the development of advanced biomaterials capable of overcoming the limitations of conventional antibiotics. In this context, metal nanoparticle hybrid hydrogels (MNHHs) have emerged as multifunctional platforms that integrate the high water-retention capacity and biocompatibility of hydrogels with the antimicrobial properties of metallic nanoparticles (MNPs). This review critically analyzes recent advances in the design, physicochemical properties, antimicrobial mechanisms, and biomedical applications of these systems. Current evidence demonstrates that MNHHs can achieve antimicrobial efficiencies above 98–99%, with minimum inhibitory concentrations as low as 0.78 µg mL^−1^ and inhibition zones of up to 25 mm against clinically relevant pathogens. Furthermore, the incorporation of MNPs significantly improves the mechanical properties of hydrogels and enables controlled and sustained metal ion release for periods of up to 14 days. Despite these promising results, important challenges remain regarding cytotoxicity, release control, the lack of experimental standardization, and the limited understanding of long-term biological effects. Overall, MNHHs represent a promising strategy for infection control, regenerative medicine, and controlled drug delivery; however, their clinical translation still requires the development of reproducible, safe, scalable, and highly biocompatible systems.

## 1. Introduction

AMR, defined as the ability of microorganisms to survive or proliferate in the presence of antimicrobial agents, is one of the most critical threats to global public health in the 21st century [1]. Global epidemiological surveillance has documented increasing levels of resistance in multiple priority bacterial pathogens, significantly compromising the effectiveness of widely used antimicrobial therapies and reducing available therapeutic options [1]. Recent projections indicate that, if current trends persist, the global burden of AMR will increase substantially in the coming decades. Deaths directly attributable to resistant infections are estimated to increase from approximately 1.14 million in 2021 to nearly 1.91 million annually by 2050 [2,3]. Overall, AMR could be associated with up to 8.22 million deaths per year, accumulating to more than 39 million deaths between 2025 and 2050 [2,3]. In addition to its health impact, AMR represents a major economic challenge, with potentially cumulative global losses approaching $100 billion in the coming decades. Among the most clinically relevant bacterial pathogens associated with this phenomenon are *Staphylococcus aureus* (*S. aureus*), *Pseudomonas aeruginosa* (*P. aeruginosa*), *Klebsiella pneumoniae* (*K. pneumoniae*), and *Escherichia coli* (*E. coli*), responsible for a significant proportion of hospital and community infections worldwide [4].

**Figure 1:**
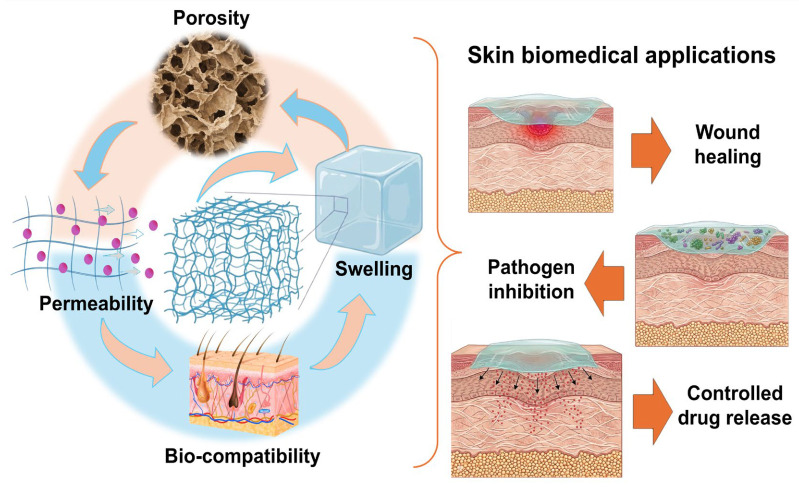
Hydrogel characteristics enable their application in skin biomedical treatments, including wound healing, pathogen inhibition, and controlled drug release.

The decline in the effectiveness of conventional antibiotics is linked to various bacterial adaptive mechanisms, including biofilm formation, horizontal transfer of resistance genes, and the emergence of mutations at antibiotic target sites. These processes reduce the penetration and activity of antimicrobial agents, promoting the persistence of chronic and recurrent infections, particularly in clinical settings under high selective pressure [4]. The increasing ineffectiveness of antibiotic treatments has driven the development of alternative therapeutic strategies aimed at improving antimicrobial efficacy and reducing systemic adverse effects. Among these strategies, functional biomaterials capable of delivering antimicrobial agents in a localized and sustained manner have emerged as promising platforms for the treatment of complex infections [5]. Among the advanced biomaterials studied in this area, hydrogels have received significant attention due to their physicochemical properties and broad range of applications. Hydrogels are 3D polymeric networks that can hold large amounts of water or biological fluid in their structure due to physical or chemical cross-links without collapsing (Figure 1). Such high water content was combined with other properties like their biocompatibility, mechanical flexibility, and similarity in structure to the native extracellular matrix (ECM), resulting in a highly attractive class of material suitable for a wide range of biomedical applications (e.g., controlled drug delivery, tissue engineering, and wound management) [6].

Although hydrogels have many advantages, traditional hydrogels possess intrinsic drawbacks that may limit their performance in addressing complicated infections. These are inherently limited in antimicrobial activity; their mechanical stability is relatively low, and the release of therapeutic agents can be controlled insufficiently [7]. To overcome these limitations, the incorporation of MNPs into the hydrogel matrix has emerged as a promising strategy, leading to hybrid systems known as MNHHs. These systems combine the structural and biocompatible properties of hydrogels with the potent antimicrobial activity of MNPs such as Ag, Cu, or Zn [7].

MNPs can act simultaneously as structural scaffolds and active antimicrobial platforms. Their mechanisms of action are multifactorial and include the generation of reactive oxygen species (ROS), capable of damaging cell membranes, proteins, and bacterial genetic material; the disruption of cell wall integrity and bacterial biofilms; the controlled release of metal ions such as Ag^+^, Cu^2+^, or Zn^2+^, which interfere with essential enzymatic processes; and the modulation of the local inflammatory response, which can promote tissue repair processes [8,9]. In particular, the incorporation of silver NPs (AgNPs) into hydrogel matrices has been shown to confer significant antibacterial activity against Gram-positive and Gram-negative bacteria [8,10]. This effect is mainly attributed to the controlled release of Ag^+^ ions and the direct interaction of the AgNPs with the bacterial cell envelope, which causes alterations in membrane integrity, inactivation of intracellular proteins, and disruptions in essential metabolic processes that lead to the inhibition of microbial growth [10]. Consistent with these mechanisms, composite hydrogels based on nanocellulose and alginates loaded with AgNPs have demonstrated bacterial reductions of nearly 99% against clinically relevant pathogens [11]. In addition to their bactericidal activity, these nanocomposites can promote tissue repair processes by reducing the microbial load at the site of injury and promoting favorable conditions for healing in experimental models of infected wounds [12].

The development of these biomaterials has also gained relevance from a technological and industrial perspective. The global market for biomedical hydrogels, particularly those intended for wound treatment, shows sustained expansion driven by the increasing incidence of chronic wounds, burns, and complications associated with antibiotic-resistant infections [12,13]. Various industry analyses project annual growth rates of approximately 7–8% during this decade, fueled by the growing demand for advanced materials for wound management and the controlled release of therapeutic agents [13]. Simultaneously, advances in green synthesis methods for MNPs, which employ plant extracts, polysaccharides, or amino acids as natural reducing and stabilizing agents, have contributed to improving the biocompatibility and environmental sustainability of these systems [14].

Nevertheless, there still remain some challenging obstacles that hinder large-scale clinical application of MNHHs. They are potentially cytotoxic due to the over-release of metal ions, the limited control on the release of MNPs from the hydrogel matrix, and the possible persistence of metal residues in the environment. Optimizing the balance between antimicrobial activity, material stability, and biosafety is therefore a key objective for the development of these systems. Thus, this review aims to provide a comprehensive and critical overview of MNHHs, addressing their physicochemical properties, antimicrobial mechanisms, and main biomedical applications. It further discusses the latest innovations in production techniques, eco-friendly synthesis processes, biosafety issues, and future clinical translation potential in the context of the emerging challenge of AMR.

## 2. Characteristics and Properties of Hydrogels

### 2.1. Design Strategies and Structural Composition of Hydrogels

Hydrogels are 3D polymeric networks assembled by hydrophilic chains, which can hold large amounts of water or biological liquids without losing their structure. This exceptional hydration capacity, combined with their soft and elastic nature, enables hydrogels to closely resemble living tissues, making them indispensable in biomedical and biotechnological applications such as wound healing, tissue regeneration, and controlled drug delivery [15,16]. The mechanical properties, swelling, and sensitivity to environmental cues are mainly determined by the polymer backbone composition and the crosslinking mechanism [17].

Hydrogels are formed from natural, synthetic, or hybrid polymers. In the case of natural polymer hydrogels, such as those based on collagen, chitosan, alginate, and hyaluronic acid, they are characterized by high intrinsic biocompatibility and biodegradability, which allow their application in direct cell contact and enzymatic degradation within the physiological milieu [18]. However, their limited mechanical stability and batch variability may restrict their long-term or load-bearing applications. On the other hand, synthetic hydrogels, including those based on poly(ethylene glycol) (PEG), poly(vinyl alcohol) (PVA), and poly(acrylic acid) (PAA), are engineered through precise polymerization and crosslinking processes that provide tunable mechanical strength, porosity, and degradation rates [19]. These artificial matrices enable precision control of the physical and chemical aspects, leading to predictable performance and production on the industrial scale. However, the application of synthetic polymers may be limited by their low biodegradability, low biological activity, and poor cellular adhesion. Nevertheless, these characteristics are intrinsic to each polymer. For instance, hydrogels based on Pluronic F127 are characterized by good mechanical properties, whereas polyacrylamide hydrogels exhibit high brittleness [20,21]. To modulate the properties of hydrogels, the development of hybrid hydrogels has emerged as an alternative strategy. These hydrogels contain both natural and synthetic components and are designed to combine the biological functionality of natural polymers with the mechanical stability and processability of synthetic material [9]. The strategy of combining both types of polymers is based on the complementary properties they provide, including the enhanced mechanical properties of synthetic polymers and the biodegradability and high biocompatibility of natural polymers. However, in the literature, the term hybrid hydrogels is used not only to describe hydrogels formed through chemical or physical cross-linking between natural and synthetic polymers but also hydrogels combined with NPs, proteins, and/or nano-/microstructures, indicating that the definition of this material remains a matter of debate. Thus, for the purposes of this review, the term hybrid hydrogels refers to hydrogels formed by combining natural and synthetic polymers. For instance, hydrogels composed of chitosan (a natural polymer) and poly(vinyl alcohol) (a synthetic polymer), cross-linked with genipin, are classified as hybrid hydrogels [22]. Another example is a hydrogel formed from poly(N-isopropylacrylamide) (a synthetic polymer) and nanocellulose (a natural polymer) [23].

Hydrogels may also be divided based on their means of crosslinking. Physically crosslinked hydrogels are held together by weak reversible forces such as hydrogen bonds, ionic interactions, and hydrophobic associations and can dynamically respond to temperature, pH, or ionic strength [24,25]. Because these interactions can dissociate under certain conditions, physically crosslinked systems often exhibit reversible swelling and self-healing behavior, making them attractive for injectable and stimuli-responsive formulations. Chemically crosslinked hydrogels are well-defined but permanent covalent crosslinks. Such hydrogels exhibit superior mechanics and structural integrity that are favorable for amaranthaceous devices or long-term drug delivery vehicles [26].

Another level of classification is based on the polymeric structure. Hydrogels may be formed from polymers containing a single type of monomer unit (homopolymeric), different types of monomer units (copolymeric), or more complex architectures known as interpenetrating polymer networks (IPNs), in which two or more polymer networks coexist without covalent bonds between them [27]. IPNs exhibit enhanced mechanical strength, controlled degradation, and multifunctionality, offering a platform for advanced biomedical applications. The swelling capacity, permeability, and biomolecule interaction of a hydrogel are also modulated by its ionic charge and crystallinity. For example, anionic hydrogels (negatively charged) favor the loading of cationic drugs, whereas cationic systems promote the adsorption of negatively charged biomolecules, such as nucleic acids and proteins, due to the electrostatic attraction between opposite charges [17]. This promotes the controlled release of the drug, which is governed by the influence of the biological microenvironment on these ionic interactions.

### 2.2. Effect of NPs on the Physical and Chemical Properties of Hydrogels

In biomedical and industrial applications, the functional performance of hydrogels is strongly determined by their physical and chemical properties, as these characteristics govern their interactions with biological systems. Among the most important physical properties are swelling behavior, mechanical strength, porosity, and viscoelasticity. These properties are strongly influenced by polymer composition, cross-linking density, and environmental conditions, such as temperature, pH, and ionic strength [16,28]. The swelling behavior of a hydrogel refers to its ability to absorb and retain water within its polymeric network until equilibrium is reached. The process is related to differences in osmotic pressure between the hydrogel and the medium external to it and is limited by the crosslinked polymer chains. Highly crosslinked networks typically exhibit reduced swelling due to limited chain mobility, whereas loosely crosslinked structures absorb more water, promoting enhanced molecular diffusion and nutrient transport [28,29]. This feature plays an important role in drug delivery and wound healing applications, as swelling affects the diffusion rates and release kinetics of therapeutic agents.

Mechanical strength is also an essential physical characteristic, which defines the resistance of the hydrogel to be compressed, bent, or stretched without fracturing. Mechanical performance depends not only on the density of crosslinking but also on the polymer type and molecular architecture. Reinforcement with nanofillers such as MNPs, nanoclays, or nanofibers can significantly enhance tensile strength and elasticity, yielding nanocomposite hydrogels capable of mimicking soft tissues and withstanding physiological loads [30,31]. This mechanical tunability is especially crucial for tissue engineering scaffolds, which need to be able to offer a suitable level of rigidity for structural support and at the same time be flexible enough to allow cell migration and proliferation.

Porosity is also an important factor affecting the transport of nutrients and biomolecules. The interconnected pores enable nutrient transport, waste excretion, and cell infiltration, all of which are necessary for cell survival and tissue integration [16]. Pore size can be tailored during synthesis through variations in polymer concentration, crosslinking time, crosslinking concentration, or the use of porogenic agents, yielding structures optimized for specific biomedical applications. The viscoelastic nature of hydrogels, as well as the possibility to show viscous and elastic responses under mechanical stress, contributes to their ability to absorb impact and then restore their shape, as is the case of biological tissues (e.g., cartilage, skin, etc.). This property is key for injectable formulations and load-bearing applications, as it ensures conformability and resilience [16,28].

Hydrogel chemistry defines their stability, degradability, and reactivity in the physiological milieu. In physically crosslinked hydrogels, the network is stabilized through reversible non-covalent bonds, providing flexibility and sensitivity to the environmental conditions. On the other hand, chemically crosslinked networks, which are crosslinked via covalent bonds, exhibit higher mechanical strength and degradation behavior that can be better controlled and hence are more appropriate for long-term implantation in the body [32,33]. Table 1 presents a comparative overview of the physicochemical characteristics of natural, synthetic, and hybrid hydrogels, highlighting key parameters such as swelling behavior and porosity, which play a central role in their performance in pathogen-related applications including antimicrobial delivery, infection control, and wound healing. These properties are strongly influenced by the chemical composition of the polymers, the crosslinking mechanism, and the internal network architecture of the hydrogel systems. Furthermore, Table 1 shows the requirements established by the USP Pharmacopeia for gel-forming hydrogels.

When comparing the swelling behavior of the different types of hydrogels, the analyzed hydrogels exhibit a wide variability in swelling capacities depending on polymer composition and NP incorporation [30]. This characteristic is mainly attributed to the interactions between polymer networks and the presence of inorganic phases, which can either restrict or enhance network expansion. For example, alginate-based nanocomposite hydrogels containing ZIF-8 NPs show swelling ratios ranging from 158% to 188%, which are lower than those observed in the unmodified alginate system (approximately 208%), indicating a restriction in network expansion due to additional interaction points introduced by the inorganic phase [34]. Similarly, alginate–poly(vinylpyrrolidone) systems demonstrate swelling values between 76% and 82%, which reflects the formation of a more compact network structure [28]. In addition, hybrid hydrogels loaded with titanium NPs (TiNPs) exhibit swelling ratios reaching approximately 300% in deionized water and decreasing to around 50% in phosphate-buffered saline due to sensitivity to ionic strength [24]. Notably, self-healing conductive hydrogels and chitosan-graft polyacrylamide nanocomposites exhibit swelling ratios around 2500%, due to the presence of highly expandable and water-affine polymeric networks [35,36]. These elevated swelling capacities are particularly beneficial in pathogen-related applications, especially in infected wound environments, where hydrogels must absorb wound exudates while simultaneously enabling the diffusion and sustained release of antimicrobial agents. However, despite these advantages, the incorporation of NPs may also restrict swelling behavior depending on the nature of polymer–particle interactions and network density [28,34].

On the other hand, some hydrogels exhibit a more controlled and gradual swelling process due to their network structure and/or surrounding conditions. Synthetic-like systems such as TiNPs-loaded hydrogels also demonstrate controlled swelling characteristics because of their sensitivity to ionic strength, which influences polymer relaxation and water uptake [24]. Furthermore, hydrogels with NPs show swelling alterations in response to the environmental changes, especially at the deionized water and phosphate-buffered saline conditions, indicating their sensitivity to the external stimulation [24]. This property makes them especially useful for antimicrobial systems and smart drug delivery platforms that can respond to infection-related environmental changes. Overall, these hydrogels provide greater predictability and tunability in swelling behavior compared with conventional systems. On the other hand, hybrid hydrogels represent an intermediate and often more advanced class of materials that combine different components within the same network. These hydrogels typically achieve variable swelling capacities along with improved functional performance. For instance, self-healing conductive hydrogels and chitosan-based nanocomposites show very high swelling values, allowing them to respond efficiently to environmental changes that often occur in infection sites [35,36].

**Table 1:** Representative types of hydrogels used in medicine and their physicochemical properties.

Hydrogel Type	Composition	Mechanism	Swelling Capacity/ Porosity	Properties	Ref.	USP Pharmacopeia Requirements of the Gel-Forming Polymer
Natural polymer-based hydrogel						
Alginate-based nanocomposite hydrogel	Alginate combined with ZIF-8 or resveratrol-loaded ZIF-8 NPs	Ionic crosslinking combined with NP encapsulation	Swelling ranges from 158% to 188%; porosity ranges from 58% to 60%	Enhanced wound healing and improved bioactivity	[34]	Alginate Assay: 90.8–106.0%; Arsenic: ≤1.5 μg/g; Lead: 10 μg/g; Microbial enumeration test: ≤10^2^ UFC for TAMC and TYMC; Specified microorganisms: Absence of *Salmonella* pecies and *Escherichia coli*; Loss on drying: ≤15.0%
Alginate structural hydrogel	Alginate with and without NPs	Ionic crosslinking	Porosity decreases from approximately 63% to approximately 59% after NP incorporation	Tunable structural and mechanical properties	[37]	
Polyelectrolyte complex hydrogel	CMC, alginate, and chitosan	Electrostatic complexation	Highly porous 3D structure with quantified porosity values reported in the study	High catalytic efficiency and structural stability	[37]	Sodium carboxymethyl cellulose Assay: 6.5–9.5% of Na; pH: 6.5–8.5 Loss on drying: ≤10.0%
Xylan-based hydrogel with Ag NPs	Xylan combined with Ag NPs	In situ NP formation	Pore size ranges from 30 to 100 micrometers	Strong antibacterial performance	[38]	Not applicable
Synthetic polymer-based hydrogel						
Hybrid TiNPs-loaded hydrogel	PEG and PVP combined with TiNPs	Chemical crosslinking	Swelling is approximately 300% in deionized water and approximately 50% in phosphate-buffered saline	Controlled drug release and periodontal tissue regeneration	[24]	Polyvinylpyrrolidone Assay: 11.5–12.8%; Residue to ignition: ≤0.1%; Lead: ≤10 ppm; Limit of aldehydes: ≤0.05%; Limit of hidrazine: ≤1 ppm; Vinylpyrrolidinone: ≤0.001%; 2-pyrrolidone: ≤3.0%; Peroxides: ≤400 ppm; Formic acid: ≤0.5%
PEGDA nanocomposite hydrogel	PEGDA incorporated with Au NPs	Photopolymerization	Highly interconnected porous network with controlled swelling behavior	Improved mechanical stability, biocompatibility, and controlled release capacity	[32]	Poly (ethylene glycol) 3350 Assay: 97.0–103.0%; Residue to ignition: ≤0.1%; Limit of ethylene glycol: ≤0.062%, Sum of diethylene glycol and ethylene glycol: and diethylene glycol: ≤0.2%, Limit of formaldehyde and acetaldehyde: ≤30 μg/g, acidity and alkalinity: 4.5–7.5, water determination: ≤1.0%,
Thermoresponsive PNIPAM-based hydrogel	PNIPAM combined with Ag NPs	Free radical polymerization and in situ NP incorporation	Temperature-dependent swelling behavior with reversible pore contraction above LCST	Thermoresponsive drug delivery and enhanced antibacterial activity	[39]	Not applicable
Polyurethane-based conductive hydrogel	Polyurethane combined with GO and metallic NPs	Chemical crosslinking and nanocomposite integration	Porous conductive network with high elasticity and moderate swelling capacity	Enhanced electrical conductivity, flexibility, and tissue engineering potential	[40]	Not applicable
PolyHEMA-based hydrogel	PolyHEMA combined with Ag NPs	Radical polymerization	Highly hydrated porous structure with controlled swelling properties	Improved antimicrobial activity and ocular biocompatibility	[41]	not applicable
Hybrid polymer-based hydrogel						
Alginate and PVP nanocomposite hydrogel	Alginate, PVP, and pomegranate seed NPs	Ionic crosslinking mediated by calcium ions	Swelling increases from 76% to 82%	Improved adsorption of heavy metals	[28]	
Self-healing conductive hydrogel	Oxidized dextran, carboxymethyl chitosan, and Ag NPs-decorated rGO	Schiff base reaction	Swelling ranges from 1500% to 1700%	Self-healing behavior, electrical conductivity, and antibacterial activity	[35]	Dextran 70 Limit of nitrogenous impurities: ≤0.01%; Limit of alcohol and Optical rotation: +195–+203° pH: 4.5–7.0; Loss on drying: ≤7.0%; Bacterial endotoxins test: ≤0.5 USP Endotoxin unit/mL; Weight average molecular weight: 63,000–77,000
Chitosan-graft polyacrylamide nanocomposite hydrogel	Chitosan, polyacrylamide, and AgNPs or Fe_3_O_4_ NPs	Free radical polymerization	Swelling ranges around 2568%	High drug loading capacity and antimicrobial activity	[36]	Chitosan Degree of acetylation: 70.0–95.0%; Residue on ignition: ≤1.0%; Lead: ≤0.5 ppm; Mercury: ≤0.2 ppm; Chromium: ≤1.0 ppm; Nickel: ≤1.0 ppm; Cadmium: ≤0.2 ppm; Arsenic: ≤0.5 ppm; Limit of iron: ≤10 ppm; Limit of protein content: ≤0.2%; Microbial enumeration test: ≤10^3^ UFC for TAMC and 10^2^ UFC for TYMC; Specified microorganisms: Absence of *S. aureus* and *Pseudomonas aeruginosa* Loss on drying: ≤5.0%

ZIF-8: Zeolitic Imidazolate Framework-8; CMC: Carboxymethyl Cellulose; PEG: Poly(ethylene glycol); PVP: Poly(vinylpyrrolidone); rGO: Reduced Graphene Oxide; AgNPs: Silver Nanoparticles; Fe_3_O_4_ NPs: Iron Oxide Nanoparticles (Magnetite); TiNPs: Titanium Nanoparticles; PEGDA: Poly(ethylene glycol) diacrylate; PNIPAM: Poly(N-isopropylacrylamide); PolyHEMA: Poly(2-hydroxyethyl methacrylate); GO: Graphene oxide.

Paralleling the swelling property, porosity is considered a key physicochemical characteristic that affects hydrogel performance, especially for familiarity with microbial, drug loading, and diffusion-related processes. Among the analyzed systems, NPs incorporation significantly affects pore size and internal architecture. For instance, alginate-based nanocomposite hydrogels exhibit porosity values ranging from 58% to 60%, which are lower than those of the pristine system (approximately 63%), suggesting partial pore occupation and the formation of a denser network [34]. Likewise, xylan-based hydrogels containing AgNPs present pore sizes between 30 and 100 μm, depending on the structural organization of the polymeric matrix [38]. On the other hand, polyelectrolyte complex hydrogels have a highly interconnected 3D network, formed by electrostatic interactions, which leads to a higher internal accessibility and mass transport [38]. Such control of the porosity is particularly beneficial in applications directed to pathogens, since the diffusion of the antimicrobial agent, the transport of nutrients, and even the infiltration of microorganisms can be regulated.

Additionally, it is important to highlight that only a few magnetic NPs have been approved by regulatory agencies worldwide. Between 1974 and 2020, a total of 25 MNP-based products were approved by the FDA and EMA for various applications, particularly in the diagnosis and treatment of cancer [42,43]. Furthermore, iron-based NPs used for the treatment of anemia are among the most extensively studied and commercially developed. These products are marketed under brand names such as Feraheme^®^, Venofer^®^, Dexferrum^®^, Ferinject^®^, Ferrlecit^®^, Hensify^®^, and Infed^®^ [44,45]. However, AgNPs, CuNPs, ZnNPs, and TiNPs are currently undergoing Phase I, II, or III clinical trials for dental applications [46].

In this context, no MNHH products have yet been approved by the FDA or EMA. This can be attributed to the stringent quality and manufacturing requirements, which demand thorough characterization of particle properties, critical quality attributes, and robust control over scale-up processes, stability, and impurities. Non-clinical challenges include understanding NP-specific biodistribution and immune responses, as well as implementing testing strategies aimed at refining, reducing, and ultimately replacing animal use in accordance with the 3Rs principles. For clinical applications, HMNP-based products must be clearly differentiated from conventional formulations through studies evaluating changes in pharmacokinetics, pharmacodynamics, and immunogenicity. In addition, environmental risk assessments should consider NP-specific persistence, aggregation behavior, and bioaccumulation potential.

## 3. Synthesis and Physicochemical Characteristics of MNPs

MNPs are among the most dynamic and revolutionary tools in modern biomedicine. Defined as particles with at least one dimension below 100 nanometers (nm), they display physicochemical behaviors that differ markedly from those of their bulk metals due to quantum confinement effects and a high surface-area-to-volume ratio, which significantly enhance their catalytic, optical, and biological reactivity [47]. These distinctive nanoscale aspects of MNPs are in close contact with biological components (e.g., membranes, proteins, nucleic acids), which take part in their interaction, allowing MNPs to be used in diagnostics, antimicrobial therapy, imaging, specific drug delivery, biosensing, and regenerative medicine [26,48,49]. The preparation of MNPs is one of the essential steps of nanotechnology, and this stage has a direct impact on their physicochemical properties, colloidal stability, and biological effectiveness. Based on the synthesis method, NPs can have different sizes, shapes, surface charges, and crystal structures, which affect their reactivity, toxicity, and ability to be used in biomedical applications. Synthesis methods are generally classified into chemical, physical, and biological (green methods) approaches, each offering distinctive advantages regarding size control, reproducibility, and environmental sustainability [34,50]. Table 2 presents examples of MNHHs, outlining their fundamental mechanisms, experimental conditions, and the balance between synthesis performance, advantages, and limitations. Each route, chemical, physical, or biological, confers certain characteristics to the NPs, such as particle size, morphology, surface charge, and yield, which subsequently dictate optical and biological properties.

Chemical approaches, including chemical reduction, sol–gel, and microemulsion methods, use reducing agents that transform metal ions into zero-valent atoms that nucleate and grow into NPs. Specifically, sodium borohydride (NaBH_4_) is commonly employed as a strong reducing as well as stabilizing agent, resulting in fast production of stable NPs with controlled sizes in the range of 10–50 nm and high surface charge [51]. Physical methods such as laser ablation, evaporation–condensation, or ball milling involve the application of external energy (heat, light, or mechanical energy) and lead to the formation of high-purity NPs without any chemical contamination [52]. In the end, green or biological synthesis presents a switch of scale for sustainable nanomaterial production. Natural extracts, which are high in polyphenols, proteins, and other phytochemicals, are used as reducing and capping agents; thus, the NPs gain biocompatibility and antioxidant capabilities. For instance, extract-mediated AgNPs often exhibit highly negative ζ-potentials (≈−49.8 to −56.1 mV), ensuring strong colloidal stability and reduced aggregation [53]. While this path is slower and cruder to size control, the low toxicity and the green nature make the route attractive for biomedical applications, including wound care and antimicrobial coatings. Advanced biomineralization techniques even employ enzymes, such as sulfite reductase, to template NP formation under mild, near-physiological conditions, yielding AuNPs with controlled size (approximately 10 nm), good uniformity, and high biocompatibility, albeit at higher cost and lower scalability [54].

The synthesis methods provide exceptional control over NPs’ incorporation, distribution, and functional performance within hydrogel systems, particularly when integrating different nanomaterials into polymeric matrices. In polyelectrolyte complex-based systems, hydrogels formed via electrostatic interactions between carboxymethyl cellulose, alginate, and chitosan allow the incorporation of multimetallic nanowires such as Pd/Au/Ag/Pt, yielding systems with uniform structure and high catalytic efficiency, including complete Cr(VI) reduction within 15 min [37]. In in situ biosynthesis approaches, the hydrogel matrix acts as both reducing and stabilizing medium, producing NPs with controlled sizes ranging from 5 to 30 nm and porous architectures between 30 and 100 µm [38]. In chemically crosslinked nanohydrogel composites, MNPs can be formed in situ within the network at physiological conditions, resulting in an enhanced bioactivity, including tumor suppression and ROS generation [48,49]. However, the stepwise synthesis processes and system complexity may bring about limitations on the scale and/or the reproducibility.

Additional techniques, including electrochemical deposition, radiochemical reduction, and functional nanocomposite integration, further expand the synthesis toolkit. Electrochemical synthesis, for example, allows the controlled growth of NPs such as RuO_2_ within conductive hydrogel matrices by adjusting current density, achieving high capacitance and long-term stability [55]. Radiochemical methods leverage γ-irradiation to induce NP formation without chemical reducing agents, producing uniform AuNPs or PtNPs while improving hydrogel elasticity and crosslinking density [56]. Functional nanocomposite approaches enable the incorporation of responsive NPs into hydrogels, promoting enhanced performance in applications such as drug delivery and biosensing [57,58].

**Table 2:** Comparison of MNHHs, advantages and limitations.

Synthesis Method	Description/Principle	Typical Conditions	Synthesis Performance	Advantages	Limitations	Ref.
Biofunctional NP-conjugated hydrogel (EGCG NPs)	Functionalization with epigallocatechin-3-gallate AgNPs for therapeutic effect.	Local administration; physiological pH	Accelerated wound healing (in vitro/in vivo); ROS scavenging; antibacterial activity	High biocompatibility; multifunctional	Complex synthesis route	[30]
Polyelectrolyte complex hydrogel + multimetallic nanowires	Formation of hydrogel via electrostatic interaction (CMC/alginate/chitosan) incorporating Pd/Au/Ag/Pt nanowires.	Crosslinking with Ca^2+^, citric acid, glutaraldehyde	100% Cr(VI) reduction in 15 min (batch); continuous operation up to 5 h with complete conversion	High catalytic efficiency; structural stability	Multistep synthesis; complex system	[37]
In situ biosynthesis of AgNPs in hydrogel matrix	Hydrogel acts as reducing and stabilizing matrix for AgNP formation from AgNO_3_.	Enzymatic catalysis (HRP/H_2_O_2_); synthesis time ≈ 1 min	AgNPs size: 5–30 nm; porous structure: 30–100 µm	Rapid synthesis; homogeneous NP distribution	Limited control over NP morphology	[38]
Chemical crosslinking + NP embedding (nanohydrogel composite)	Integration of Cu-based or Se-based NPs into hydrogel via CuCl_2_ crosslinking.	Physiological conditions; laser irradiation (photothermal)	Significant tumor suppression in vivo; high ROS generation; enhanced cellular uptake	Synergistic chemo-photothermal therapy	Requires external stimulation (laser)	[49]
Electrochemical deposition of RuO_2_ NPs in conductive hydrogel	Electrochemical growth of RuO_2_ NPs on rHGO/f-MWCNT hydrogel matrix.	H_2_SO_4_ electrolyte; 1 mA cm^−2^ current density	Capacitance: 480 mF cm^−2^; 93.89% retention after 10,000 cycles; energy density: 30.68 µWh cm^−2^	High electrochemical performance; stability	Limited biomedical application	[55]
Radiochemical reduction (γ-irradiation)	Formation of AuNPs/PtNPs inside hydrogel via γ-radiation without chemical reducers.	Co-60 γ irradiation	Uniform NP formation; improved elasticity and crosslinking density	Clean method; no toxic reagents	Requires radiation facilities	[56]
Ni NP-modified conductive hydrogel (biosensor)	Incorporation of NiNPs into PVA/PEDOT:PSS hydrogel for glucose sensing.	Flexible hydrogel system; dynamic strain conditions	High sensitivity and clinically relevant glucose detection limits	Wearable biosensor applications	Long-term stability concerns	[57]
NIR-responsive nanocomposite hydrogel (MSN-based)	MSNs NPs incorporated into HA hydrogel for on-demand drug release.	NIR laser stimulation; drug loading >90%	Controlled drug release under NIR; enhanced antitumor activity (HT-29 cells)	Triggered release; high loading capacity	Requires external NIR source	[58]
Keratin-based hydrogel + CuNP/NCQD nanocomposite	Biomass-derived keratin hydrogel functionalized with CuNPs and carbon quantum dots.	Ambient conditions; biodegradable matrix	>50% mass loss after 45 days; enhanced antimicrobial activity vs. *E. coli* and *S. aureus*	Sustainable; biodegradable	Moderate mechanical strength	[59]
Enzymatic crosslinking hydrogel + plasmonic NPs (SERS platform)	Incorporation of AuNPs/AgNPs in silk fibroin hydrogel for Raman detection.	Enzymatic crosslinking; excitation at 785 nm	Detection limits: 0.17–0.27 µM (hydrogel) vs. 1.56–15.63 µM (solution); signal enhancement >400×	Ultra-high sensitivity; analytical applications	Specialized instrumentation required	[60]

MNPs: Metallic NPs; AgNPs: Silver NPs; AuNPs: Gold NPs; CuNPs: Copper NPs; RuO_2_ NPs: Ruthenium dioxide NPs; NiNPs: Nickel NPs; MSNs: Mesoporous silica NPs; CMC: Carboxymethyl cellulose; HA: Hyaluronic acid; HRP: Horseradish peroxidase; H_2_O_2_: Hydrogen peroxide; ROS: Reactive oxygen species; NIR: Near-infrared radiation; rHGO: Reduced highly oxidized graphene oxide; f-MWCNTs: Functionalized multi-walled carbon nanotubes.

MNPs can be broadly classified into noble metals, base metals, metal oxides, and multifunctional nanocomposites, each offering distinctive properties relevant to biomedical use. Noble MNPs, primarily AgNPs and AuNPs, are characterized by their chemical stability, versatile integration into hydrogel matrices, and potent biological activity. AgNPs have been extensively studied for their broad-spectrum antimicrobial properties against bacteria such as *E. coli* and *S. aureus* [29,61]. Their mode of action involves the release of Ag^+^ ions and the generation of ROS, highly reactive molecules that damage microbial cell membranes and metabolic pathways [29,61]. In contrast, AuNPs are highly biocompatible and exhibit unique optical properties that enable signal amplification in sensing platforms. This property confers distinctive detection capabilities, allowing AuNPs to be used in biosensing applications where ultra-sensitive limits down to 10^0^ fg mL^−1^ can be achieved for biomolecule detection [62].

Base MNPs, such as CuNPs, possess strong antimicrobial and catalytic activities but are more susceptible to oxidation than noble metals. They generate metal ions that disrupt microbial enzymes and cell structures but can also trigger oxidative stress if not properly stabilized. To mitigate this, CuNPs are often incorporated into hydrogel matrices, which regulate ion release, improve stability, and enhance biocompatibility, particularly under synergistic conditions such as near-infrared irradiation that promote photothermal effects [63].

Metal oxide NPs, notably zinc oxide-based systems, represent multifunctional systems combining structural robustness with bioactivity. ZnONPs incorporated into hydrogels exhibit dual antibacterial and regenerative functions: they enhance antimicrobial activity, improve mechanical properties, and contribute to tissue regeneration processes such as angiogenesis and collagen deposition [64]. The biomedical performance of MNPs is thus dictated by their size, morphology, and distribution within the hydrogel network. Particles at the nanoscale tend to exhibit greater interaction with biological systems and enhanced functional performance but also require proper stabilization to avoid aggregation. Spherical NPs are commonly favored for their uniformity and stability, while more complex nanocomposite systems allow improved functionality and surface interactions for advanced biomedical applications [31,38].

## 4. Hydrogels as Matrices for MNHHs: Manufacturing, Scalability and Properties

### 4.1. Fabrication Methods

**Figure 2:**
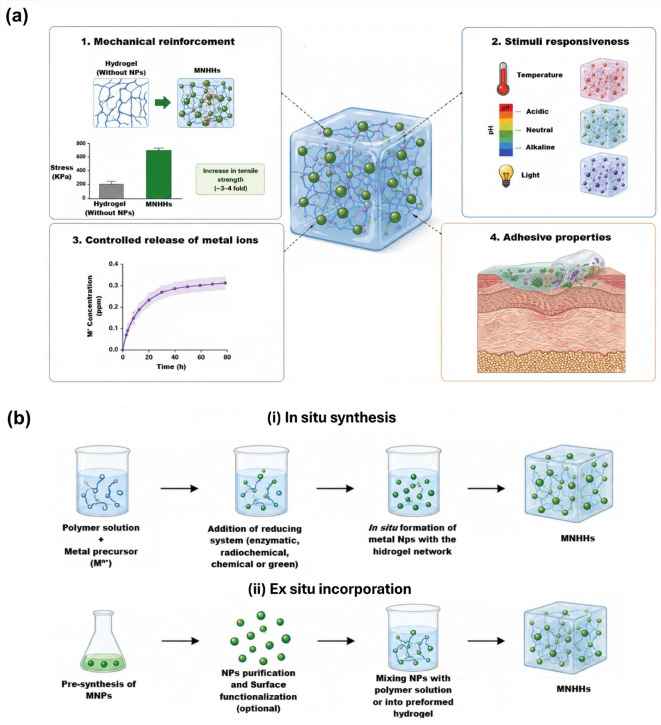
(**a**) Structural and functional advantages of MNHHs. MNPs act as secondary cross-linking points within the hydrogel network, improving stress distribution, tensile strength, elasticity, and overall mechanical stability. In addition, MNHHs exhibit stimuli-responsive behavior to external stimuli, promote sustained ion release, and display strong adhesion to biological tissues. (**b**) Synthesis strategies for MNHHs: (i) in situ synthesis and (ii) ex situ incorporation.

A major technological advancement in this field has been the incorporation of MNPs into polymer networks, leading to the development of nanocomposite hydrogels with enhanced structural and functional performance [16,38]. AgNPs, ZnONPs, CuNPs, and other multifunctional NPs act as nanofillers that occupy the intermolecular spaces within the hydrogel network, thereby enhancing its mechanical integrity. In addition, the release of metal ions provides antimicrobial activity against a broad range of pathogens, while improved adhesive properties facilitate application to skin surfaces. Depending on their composition, these hydrogels may also undergo conformational changes in response to external stimuli, such as pH, light, and temperature, among others [59,64] (Figure 2a). When uniformly distributed, these NPs function as secondary cross-linking centers that redistribute mechanical stress and enhance the durability of the material [16,38]. However, it has been demonstrated that the properties of MNHHs depend on the fabrication method.

The fabrication of MNHHs presents a multidimensional challenge that combines aspects of molecular design, control of NP dispersion, and adaptability to larger scales. The recent literature shows that NP incorporation methods affect not only the mechanical and physicochemical properties of hydrogels but also their reproducibility and clinical viability [65,66]. Two primary strategies are commonly employed to obtain MNHHs: in situ synthesis and ex situ incorporation (Figure 2b). These approaches differ in the sequence of NP formation relative to hydrogel network assembly, influencing particle dispersion, interfacial bonding, and release behavior [38,57].

Table 3 presents representative MNHHs prepared by in situ synthesis and ex situ incorporation, which are described below. In the in situ synthesis method, MNPs are generated within a forming or preformed hydrogel matrix. Typically, a polymer solution containing metal precursors is exposed to a reducing environment, which converts metal ions into zero-valent MNPs directly within the hydrogel network [38,56]. A comparative analysis of the available studies suggests that in situ methods, in which NPs are synthesized during hydrogel formation, tend to promote more homogeneous dispersion and more stable integration within the polymer matrix. For example, Al-Hussain et al. (2025) highlighted that the simultaneous formation of NPs and the polymer network allows for precise control over morphology and prevents NP aggregation, thereby improving controlled drug release [66]. However, these methods have scalability limitations, particularly when attempting to reproduce the uniformity achieved at the laboratory scale under industrial production conditions, which may restrict their applicability beyond the prototype stage [67].

Ex situ methods involve the synthesis of NPs prior to their incorporation into the hydrogel matrix, providing better control over particle size and physicochemical modifications [66,76]. However, studies indicate that this method may face challenges related to NP aggregation and non-uniform dispersion, factors that can directly affect the biological and mechanical performance of the hydrogel [65,77]. In comparison, although the ex situ method allows for NP standardization and characterization, it often requires additional strategies, such as dispersing agents or chemical functionalization, to achieve a level of homogeneity comparable to that obtained through in situ approaches [65]. Based on this critical analysis, this section is organized into two subcategories: in situ fabrication and ex situ fabrication.

#### 4.1.1. In Situ Manufacturing of MNHHs

In situ fabrication involves generating MNPs directly within the hydrogel matrix during polymerization or self-assembly, thereby promoting homogeneous dispersion and intimate interactions between the polymer network and the NPs [65,67]. This strategy has been extensively studied in polysaccharide and zwitterionic-based hydrogels and stands out for its ability to control NP morphology and promote structural integration within the hydrogel matrix [78].

The formation of NPs within the hydrogel matrix can be induced through enzymatic systems (e.g., HRP/H_2_O_2_) or radiochemical approaches, such as γ-irradiation, which generate reactive reducing species capable of converting metal ions into zero-valent NPs [38,56]. This one-pot synthesis approach promotes homogeneous NP distribution and strong interfacial adhesion between the metallic phase and the polymer chains, thereby minimizing aggregation and leaching. The hydrogel acts as a nanoreactor, in which the polymeric network confines nucleation and growth, resulting in uniformly dispersed NPs that are typically 5–50 nm in diameter [38]. For example, AgNPs synthesized in situ within hydrogel matrices through enzymatic catalysis can be obtained in approximately 1 min, with particle sizes ranging from 5 to 30 nm and a homogeneous distribution throughout porous structures measuring 30–100 µm. These characteristics contribute to improved functional performance [38]. For example, Al-Hussain et al. (2025) reported that chitosan hydrogels incorporating in situ-generated AgNPs exhibited an average particle size of 12–18 nm, with a uniform distribution throughout the matrix. This homogeneous distribution resulted in sustained drug release over 7–10 days while maintaining antibacterial activity greater than 90% against *E. coli* and *S. aureus* [66]. In comparison, hydrogels in which the NPs were incorporated ex situ exhibited partial NP aggregation, resulting in a reduction in active surface area and biological efficacy [76].

Furthermore, in situ synthesis represents a sustainable fabrication strategy, as biopolymers can serve simultaneously as structural matrices and stabilizing agents [38,59]. Natural polymer-based hydrogels have demonstrated the ability to support NP formation under mild conditions while maintaining high biocompatibility and reducing the need for additional chemical reagents. Despite these advantages, in situ fabrication requires careful control of reaction kinetics to prevent NP aggregation and ensure complete reduction of metal ions [57]. Parameters such as temperature, pH, precursor concentration, and reaction time influence NP nucleation (the formation of atomic clusters) and growth (their expansion into stable NPs). Rapid reduction may lead to the formation of irregular aggregates, whereas controlled conditions favor more uniform NP distribution. Therefore, optimizing these parameters is essential to ensure reproducibility and consistent functional performance [57]. Recent studies have also highlighted that in situ manufacturing enables the optimization of NP–polymer interactions through adjustments in pH, temperature, and reaction time [65,66]. In zwitterionic hydrogels, in situ NP synthesis confers self-healing properties and enhances resistance to mechanical degradation, increasing the elastic modulus by up to 30% compared with matrices lacking NPs [65]. Furthermore, direct integration during polymerization reduces the need for dispersing agents, thereby simplifying the fabrication process and decreasing the risk of additive-induced cytotoxicity [66].

From a scalability perspective, Ali et al. (2025) point out that, although in situ synthesis improves homogeneity and reproducibility at small scales, its industrial-scale implementation faces significant challenges. These include achieving a homogeneous distribution of NPs in large-volume systems, managing reaction heat during polymerization, and controlling variability in cross-linking processes, which can affect the mechanical properties of the hydrogel and the release of therapeutic agents [67]. Andjela et al. (2025) also highlight that, in NP-enhanced photopolymerization processes, controlling exposure time and NP concentration is critical to preventing aggregation and ensuring the structural fidelity of hydrogel microarchitectures [77]. In general, it has been shown that in situ synthesis is more favorable for hydrogels in biomedical applications, where matrix integrity and controlled drug release are particularly important. In contrast, the ex situ approach may offer advantages in the precise characterization of individual NPs; however, it can negatively affect the final homogeneity of the material [76].

#### 4.1.2. Ex Situ Manufacturing of MNHHs

Ex situ fabrication involves synthesizing MNHHs independently and then incorporating them into the hydrogel matrix. This approach allows for more precise control over the size, morphology, and composition of the NPs, which is particularly relevant when reproducibility is required in advanced biomedical applications [76,79]. Pre-synthesized NPs are fabricated separately using different synthesis routes and then incorporated into the hydrogel matrix. This sequential process allows independent optimization of NP size, morphology, and surface functionalization before embedding [55,57].

NPs can be mixed into the polymer precursor solution prior to gelation or introduced into preformed hydrogels via diffusion or electrostatic interactions [55]. For example, Montero et al. (2025) demonstrated that iron and Fe_3_O_4_ NPs synthesized by recycling steel waste exhibited controlled sizes between 50 and 125 nm, with a saturation magnetization of up to 200 emu/g. The ex situ incorporation of these NPs into polysaccharide hydrogels preserved the magnetic and structural integrity of the matrix, facilitating potential applications in targeted drug delivery and magnetic biosensors [80]. Similarly, Kumari et al. (2025) reported that the ex situ addition of MNPs to cellulose hydrogels significantly improved their mechanical and conductive properties, enabling the fabrication of scaffolds for tissue engineering and advanced wound dressings [76].

Additionally, ex situ incorporation has been applied to produce conductive hydrogel systems through the incorporation of NiNPs or RuO_2_ NPs, demonstrating advanced performance in biosensing and electrochemical applications, with capacitance values of up to 480 mF cm^−2^ with 93.89% retention after 10,000 cycles and energy densities of 30.68 µWh cm^−2^ [55,57]. Likewise, this method has also been applied to controlled drug release. For instance, MNHHs incorporating mesoporous SiO_2_ NPs enable anticancer drug loading efficiencies greater than 90% and controlled release under near-infrared (NIR) stimulation, thereby enhancing therapeutic performance [58]. However, ex situ incorporation faces challenges related to dispersion and stability. NPs often aggregate during mixing due to interparticle interactions [57]. To prevent this, surface functionalization with hydrophilic ligands is commonly employed to enhance colloidal stability and interfacial compatibility [55,57]. Ultrasonic treatment and mechanical stirring are also used to promote uniform dispersion. Notably, NP loading strongly affects the mechanical behavior of hydrogels: moderate concentrations enhance strength and functionality, whereas higher loadings may induce structural instability or heterogeneity, as observed in biodegradable systems with mass losses greater than 50% after 45 days [59].

In terms of scalability, ex situ manufacturing offers advantages over in situ synthesis, particularly when large quantities of NPs with reproducible characteristics are required [67,77]. However, this method can increase process complexity due to the need for additional purification, stabilization, and matrix compatibilization steps and may affect the final homogeneity of the hydrogel if the incorporation parameters are not properly controlled. In 3D bioprinting applications, Pavithra et al. (2025) demonstrated that the ex situ integration of polymeric NPs into bioinks improves structural fidelity and mechanical strength but requires precise adjustments to viscosity and rheology to maintain printability and cell viability [79]. Compared to in situ synthesis, ex situ fabrication prioritizes reproducibility and control of the intrinsic properties of NPs, while potentially compromising dispersion homogeneity and intimate interaction with the polymer network.

### 4.2. Production Costs and Scalability

A complete assessment of the technical viability and potential for economic and industrial scale-up of MNHHs as described in the recent literature is warranted. Within this frame, production cost is influenced by three main elements: (i) the type of NP, (ii) the method of synthesis, and (iii) the level of complexity of the polymer system of the hydrogel [65]. Regarding raw materials, zinc oxide (ZnO) stands out as one of the most economically accessible nanomaterials. Its price can range from approximately 10 to 100 USD/kg, depending on purity, particle size, and purchase volume [81,82]. This relative affordability favors its implementation in scalable biomedical systems.

At the market level, zinc nanooxide reached a value of USD 352.1 million in 2023 and is projected to reach USD 865.6 million by 2032, with a compound annual growth rate close to 11% [83]. This sustained growth is driven by its broad applicability in the biomedical, cosmetic, electronics, and coatings sectors, as well as by the existence of multiple established synthetic routes, such as sol-gel, hydrothermal, and chemical deposition methods [83]. Furthermore, the global availability of zinc, with an approximate production of 13 million metric tons per year, supports a robust and scalable supply chain [83]. However, environmental and toxicological concerns associated with ZnO could increase regulatory costs in the future [66].

In contrast, noble MNPs, such as AgNPs and AuNPs, have considerably higher costs due to both the price of the base metal and the complexity of their synthesis processes [84,85]. The global AgNP market reached approximately USD 4.2 billion in 2025 and is projected to reach USD 16.8 billion in 2035, with a compound annual growth rate of 14.8% [85]. This growth is primarily driven by applications in the healthcare, advanced electronics, and antimicrobial textiles sectors. However, its production remains expensive due to the volatility of the metal’s price, which can impact between 15 and 20% of the total cost, as well as stringent regulatory requirements [85].

From a process perspective, green synthesis strategies and rapid polymerization methods are emerging as key alternatives for reducing operating costs. NP biosynthesis and biogenic-mediated in situ formation eliminate the need for expensive reagents and energy-intensive conditions [68,78]. Similarly, approaches such as deep eutectic solvent (DES)-assisted polymerization enable the fabrication of hydrogels in short timeframes and without the use of toxic initiators, significantly reducing production costs and facilitating scalability [72].

Another determining factor is the efficiency of NP use. Reusable catalytic systems, such as chitosan hydrogels with bimetallic CuAuNPs, demonstrate that it is possible to achieve high functional activity using low metal loadings, improving the cost-benefit ratio [74]. Similarly, the design of multifunctional MNHHs allows for the integration of multiple properties into a single matrix, reducing processing steps and associated costs [72]. In terms of scalability, both the zinc nanooxide and MNPs markets show sustained growth trends driven by their applications in health, electronics, and advanced materials [83,84]. The expansion of production, along with process automation and advanced quality control, is driving the transition to large-scale production. However, challenges related to reproducibility, particle size control, stability, and regulatory compliance persist, especially in biomedical applications, which can increase overhead costs and limit mass adoption [68,85].

Overall, ZnO-based systems currently offer the best cost-scalability ratio due to their low cost, availability, and industrial maturity, while noble metal-based nanomaterials offer greater functional performance at the expense of higher costs and production complexity [83,85]. However, market expansion, along with advances in sustainable synthesis and process optimization, suggests that these limitations could be mitigated in the medium term [68,72]. In this context, the integration of green approaches, efficient processes, and multifunctional designs emerges as the most promising strategy for the development of economically viable and industrially scalable nanocomposite hydrogels.

### 4.3. Physicochemical Properties and Controlled Release of Ions of MNHHs

The incorporation of MNPs into hydrogel matrices produces multifunctional nanocomposites with superior physicochemical and biological properties. This reinforcement arises from the interfacial interactions between the NPs and the polymeric network, which alter crosslinking density, porosity, and mechanical integrity [16,38]. In these systems, MNPs act as nanofillers that restrict polymer chain mobility, thereby improving mechanical strength, toughness, and elasticity, while simultaneously providing active antimicrobial or catalytic functionality [59,64].

From a structural standpoint, the addition of NPs increases network heterogeneity, leading to modifications in pore architecture and enhanced load-bearing capacity. Recent studies have shown that the incorporation of ZnONPs into hydrogel matrices can improve mechanical stability through ion-mediated crosslinking mechanisms, without compromising biocompatibility or functional performance [86]. This improvement is associated with interactions between NP surfaces and the functional groups (–NH_2_, –COOH, –OH) of the polymer chains, which contribute to a more integrated and stable network structure. Similarly, hydrogels incorporating NP systems such as Ag-loaded mesoporous silica have demonstrated dynamic structural reorganization in response to environmental stimuli, enhancing both mechanical adaptability and biological functionality [87].

In addition to mechanical reinforcement, MNPs confer functional enhancements such as electrical conductivity, thermal stability, and bioactivity. For instance, electrochemically decorated RuO_2_ NPs incorporated into conductive hydrogel systems based on reduced graphene oxide and carbon nanotubes create highly conductive 3D networks suitable for energy storage applications. These systems exhibit remarkable electrochemical performance, reaching capacitance values up to 480 mF cm^−2^ and maintaining high stability over repeated charge–discharge cycles, highlighting their potential for advanced electrochemical and bioelectronic applications [55]. Similarly, AgNPs incorporated into hydrogel systems provide enhanced antibacterial and antibiofouling properties, enabling their application in biomedical devices such as urinary catheters. These systems, based on microcapsule-loaded hydrogels, allow sustained release of silver ions and demonstrate high antibacterial efficiency (>99.99% against common pathogens), along with improved mechanical performance, lubricity, and biocompatibility [30]. These multifunctional properties have broadened the biomedical applications of MNHHs to include smart wound dressings, on-demand drug delivery systems, and stimuli-responsive scaffolds [59,87]. Systems incorporating MNPs, such as AgNP, ZnONP, or MgFe_2_O_4_, show significant mechanical reinforcement. For example, Mei et al. (2026) have a compressive strength of 25 kPa and a Young’s modulus of 1.8 MPa, and the FA-Ag/CPH systems achieve a compressive strength of 28 kPa [88]. In comparison, hydrogel systems incorporating NP-loaded microcapsules demonstrate enhanced mechanical stability and functional performance, as observed in PVA-based hydrogels embedded with AgNP-loaded alginate–chitosan microcapsules, which exhibit notable tensile strength (~0.16 MPa) along with improved antibacterial and antibiofouling properties [30].

The swelling behavior of MNHHs is another critical physicochemical characteristic influencing metallic ions’ diffusion-controlled processes. NPs’ incorporation typically reduces equilibrium swelling due to increased network rigidity and reduced free volume, as observed in alginate-based nanocomposite hydrogels where swelling decreases from approximately 208% in pristine systems to 158–188% after NP incorporation [34]. Nevertheless, maintaining adequate hydration (above 70% water content) remains feasible through controlled NPs loading and surface modification using hydrophilic stabilizers, such as poly(ethylene glycol) or polysaccharides [48,49]. This balance between stiffness and water retention is essential for biomedical performance, particularly in wound care and tissue engineering applications. For instance, MNHHs are considered effective for the controlled retention of metal ions, as in hydrogels with AgNPs that release only ~0.35 ppm in 7 days, allowing for sustained antimicrobial activity without inducing significant cytotoxicity [32]. This balance between stability and functionality is key in applications such as wound healing, where prolonged and biocompatible action is required [61].

MNPs also significantly influence the thermal and oxidative stability of hydrogels. The incorporation of NPs enhances structural integrity and resistance to degradation processes, improving the long-term stability of the hydrogel network under physiological and operational conditions. For example, nanocomposite hydrogels incorporating MNPs have demonstrated remarkable stability, maintaining up to 93.89% capacitance retention after 10,000 electrochemical cycles, reflecting strong resistance to structural degradation [55]. Similarly, AgNP–based hydrogel systems exhibit sustained antibacterial activity and preserved structural performance over extended periods, maintaining functional properties even after 60 days of exposure, which indicates enhanced resistance to environmental and oxidative stress [30]. In summary, the physicochemical reinforcement of hydrogels with MNPs provides an effective strategy to synergistically integrate mechanical durability, environmental responsiveness, and antimicrobial performance. These enhancements establish MNPHs as next-generation biomaterials that bridge the gap between soft matter mechanics and nanotherapeutic functionality, forming the foundation for the following section on their biological interactions and therapeutic performance.

On the other hand, Table 4 shows the different conditions and ion release from MNHHs, allowing visualization of how the composition of the hydrogel and the nature of the nanocomponents influence the effectiveness of the material, highlighting those designs that combine structural integrity with release controlled by agents bioactive, optimizing both functionality and antibacterial properties such as biocompatibility. Metallic incorporation impacts the functionality, stability, and controlled release of metallic ions until 14 days for Ag^+^, indicating an efficient material for biomedical applications. Other reports with more rapid releases also have been reported. For instance, AgNPs of 15 nm in multilayer hydrogels formed by Yan et al. (2021) enabled a pH-dependent, stepwise release pattern, releasing approximately 70% of the active content within 48 h [89]. ZnO/DAT (decellularized Achilles tendon) systems released 0.25% Zn^2+^ in a sustained manner for 72 h, promoting proliferation and tenogenic differentiation of TDSC cells [86]. Even these systems have been used to modulate the release of active compounds, founding both controlled release of the ions and drugs [90].

## 5. Applications of MNPs and MNHHs for the Inhibition of Clinical Pathogens

### 5.1. Antimicrobial Mechanisms of MNPs

**Figure 3:**
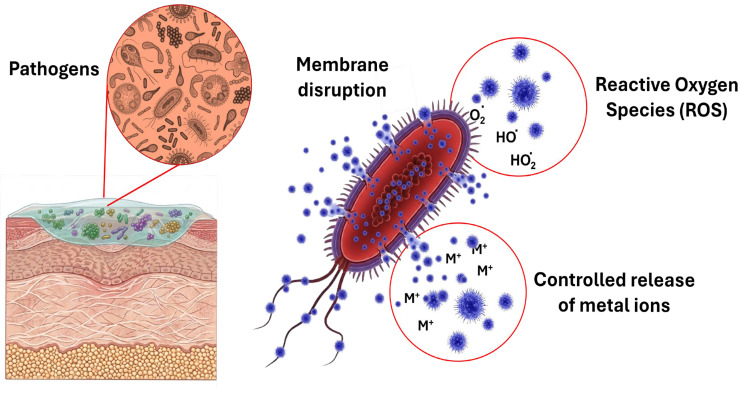
Mechanisms of action of MNPs against clinical pathogens include their attachment to the cell membrane, leading to structural disruption and increased permeability, which results in the leakage of cytoplasmic contents. Additionally, MNPs generate ROS that induce oxidative stress, causing damage to proteins, lipids, and nucleic acids within the pathogen. The controlled release of metal ions (e.g., Ag^+^, Cu^2+^) further inhibits enzymatic activity and interferes with essential cellular processes.

The antimicrobial potential of MNPs arises from complex, interrelated mechanisms that compromise microbial viability, inhibit proliferation, and interfere with essential cellular functions. Unlike conventional antibiotics that target specific metabolic pathways, MNPs act through multiple physicochemical and biochemical routes simultaneously, producing broad-spectrum activity and minimizing the risk of resistance development [32,63]. Figure 3 shows the antimicrobial pathways. MNPs attach to microbial membranes and most significantly include direct membrane interaction, the generation of ROS, and the controlled release of metal ions such as Ag^+^, Cu^2+^, and Au^3+^, each contributing to a multifaceted antimicrobial effect [30,63,64].

A primary mechanism involves direct interaction of MNPs with microbial cell membranes. The surfaces of most bacterial and fungal cells carry a negative charge due to the presence of teichoic acids and phospholipids, allowing electrostatic attraction to the typically positive or cationic surfaces of MNPs [32,64]. This interaction causes structural disorganization of the lipid bilayer, formation of pores, and leakage of cytoplasmic contents, ultimately leading to cell lysis. The small particle size of MNPs (<20 nm) facilitates their insertion into the membrane, enhancing permeability and accelerating microbial death. Studies have shown that AgNPs and AuNPs within the range of 10–20 nm exhibit significantly lower minimum inhibitory concentrations (MICs) compared to larger particles, confirming that nanoscale dimensions increase antimicrobial potency [62]. Another major pathway of antimicrobial activity is the generation of ROS, including hydroxyl radicals (•OH), superoxide anions (O_2_^−^), and hydrogen peroxide (H_2_O_2_). These highly reactive molecules oxidize lipids, proteins, and nucleic acids, resulting in irreversible cell damage and microbial apoptosis or necrosis [48,49,63]. For instance, ZnO and α-Fe_2_O_3_ NPs have been shown to reduce *P. aeruginosa* biofilm viability by more than 80% through oxidative stress and penetration into biofilm matrices [63]. The ROS-mediated damage extends to mitochondria-like structures in fungi and to viral envelopes, leading to loss of metabolic activity and infectivity.

A third and complementary antimicrobial route involves the release of metal ions. During their gradual oxidation, NPs release ions such as Ag^+^, Cu^2+^, or Au^3+^ that diffuse through microbial membranes and bind to thiol (–SH), amino (–NH_2_), and carboxyl (–COOH) groups in proteins and enzymes [32,94]. This interaction disrupts electron transport chains, inhibits ATP production, and leads to protein denaturation. Ion binding to nucleic acids can also prevent DNA replication and transcription, further inhibiting cell division [48,49]. Because these processes act simultaneously, the combined effect of ROS generation and ion release amplifies the bactericidal outcome and significantly reduces the likelihood of resistance.

The incorporation of MNPs into hydrogel matrices introduces an additional level of control and biocompatibility. Hydrogels provide a hydrated, porous environment that supports sustained NPs release while maintaining localized antimicrobial activity at the infection site [30,31]. Their high water content and soft mechanical properties ensure intimate contact with tissues, promoting efficient delivery of metal ions and reactive species while limiting systemic exposure. Moreover, the polymeric matrix minimizes NPs aggregation, ensuring homogeneous dispersion and consistent antimicrobial performance [15,95]. The interplay of these mechanisms results in a multifactorial antimicrobial response that includes membrane disruption, oxidative stress, and metabolic inhibition. For example, AgNPs-loaded chitosan hydrogels demonstrated over 90% inhibition of *S. aureus* and *E. coli* growth within 24 h, attributed to the combined effects of Ag^+^ ion release and polymer-mediated adherence to bacterial surfaces [34]. Likewise, AuNPs-gelatin hydrogels showed strong antiviral effects by damaging viral envelopes and preventing host-cell binding [96], while CuNPs-based systems inhibited *Candida albicans* biofilm formation by suppressing hyphal growth and ECM synthesis [97].

The physicochemical features of both the NPs and the hydrogel influence antimicrobial efficacy. Factors such as NP size, surface charge (ζ potential), and hydrogel porosity determine the rate of ion diffusion, ROS production, and penetration into microbial biofilms [95]. Smaller NPs with ζ potentials exceeding ±30 mV exhibit enhanced colloidal stability, improving uniform distribution in the hydrogel and prolonging antimicrobial activity [15,31]. The synergistic optimization of these parameters enables tunable antimicrobial performance suited for various biomedical applications such as wound dressings, implant coatings, and infection-resistant scaffolds. In conclusion, the antimicrobial activity of MNPs and their hydrogel composites is characterized by a convergence of physical and chemical effects that disrupt microbial homeostasis at multiple levels. Their combined mechanism of membrane damage, oxidative stress, and ion toxicity offer a broad and efficient antimicrobial defense, making these nanocomposite hydrogels promising candidates for infection control, wound healing, and therapeutic coatings in biomedical applications.

### 5.2. MNHHs Against Bacterial Infections

MNHHs constitute a highly versatile and promising class of antimicrobial biomaterials that combine the biocompatibility, hydration, and mechanical softness of hydrogel matrices with the potent microbicidal properties of MNPs, such as AgNPs, AuNPs, and CuNPs [68,71]. While the antimicrobial mechanisms of MNPs, namely membrane disruption, ROS generation, and metal ion release, have been described in Section 5, embedding NPs within a hydrogel matrix adds multiple advantages by modulating their release and interactions with microbial cells. The hydrogel network not only enables localized and sustained delivery of bioactive ions and ROS but also enhances colloidal stability, reduces systemic toxicity, and provides a protective environment that prolongs NP activity [31,92]. These combined features allow MNHHs to inhibit a wide range of pathogens, including Gram-positive and Gram-negative bacteria, more effectively and for longer periods than conventional antibiotics [98,99].

The hydrogel matrix itself plays a critical role in determining antimicrobial efficacy. Its porosity and crosslinking density govern the diffusion of ions and ROS, thereby controlling the kinetics of microbial inhibition. Highly crosslinked hydrogels restrict NP migration, favoring prolonged release suitable for sustained antimicrobial action, whereas more loosely crosslinked hydrogels permit faster diffusion, which may be advantageous for acute infection control [92]. Furthermore, the physicochemical characteristics of NPs, such as particle size and surface charge, critically influence their dispersion within the hydrogel and the consistency of antimicrobial effects. In this context, green or biological synthesis has been increasingly explored, as natural extracts rich in polyphenols and other phytochemicals act as reducing and stabilizing agents [92]. For instance, extract-mediated NPs exhibit highly negative ζ-potentials (≈−49.8 to −56.1 mV), ensuring strong colloidal stability and reduced aggregation [53]. These characteristics favor improved NP dispersion within hydrogel matrices and more consistent antimicrobial performance, while surface functionalization with polymers such as chitosan further enhances stability and interactions with microbial membranes [100].

Table 5 describes several representative studies of MNHHs, including NP type, hydrogel carrier, particle size, target pathogens, MIC, exposure time, mechanism of action, and observed outcomes. Among the NPs, AgNPs remain the most studied and effective antimicrobial agents. Their incorporation into hydrogels such as chitosan or PVA enables the inhibition of both Gram-positive and Gram-negative bacteria. For instance, AgNP-based hydrogels exhibited inhibition zones ranging from 9 to 19 mm against *E. coli*, *S. aureus*, *P. aeruginosa*, and *K. pneumoniae* [68]. Similarly, AgNP thermosensitive hydrogels achieved a MIC of 25 µg/mL, resulting in approximately 95% inhibition after 24 h exposure [12]. The observed antimicrobial effect is primarily mediated by the release of Ag^+^ ions, which interact with bacterial cell membranes and induce oxidative stress (ROS), causing protein denaturation and DNA damage. Another study has reported that AgNP-based hydrogels exhibit >99.99% inhibition against *S. aureus* and *E. coli* while maintaining inhibition zones of approximately 16 mm, highlighting their strong antibacterial performance [30]. Also, AgNP systems showed an effect over time; for instance, controlled-release hydrogels presented silver loading of 122.6 mg/g and release levels of 33–52 µg/L, enabling prolonged antibacterial activity [92].

Moreover, particle dispersion and physicochemical characteristics were critical determinants of activity, as well-dispersed NPs within hydrogel matrices exhibited consistent antimicrobial effects. For example, ZnO NP-based hydrogels achieved a MIC ≈ 1.95 µg/mL against *P. aeruginosa* and *S. epidermidis*, evidencing strong antimicrobial performance associated with ROS generation and cell wall disruption [101]. Similarly, IONP-based hydrogels demonstrated MIC values of 0.78–1.25 µg/mL against *A. baumannii*, indicating enhanced activity against resistant strains [39].

On the other hand, CuNPs showed broad-spectrum activity at concentrations of 30–50 mg/mL, effectively inhibiting both *P. aeruginosa* and MRSA, with the mechanism attributed to ion release, ROS generation, and membrane destabilization [71]. Other reports with CuNP hydrogels have been employed for antibacterial therapy, achieving bacterial reductions of up to 10^6^ CFU/mL, attributed to combined photothermal and ion-release effects [63]. In addition, hybrid formulations such as GO–Ag hydrogels demonstrated synergistic effects, producing inhibition zones of up to 39 mm, surpassing conventional systems due to combined ROS generation and membrane disruption [98]. In addition, the synergy between MNPs and hydrogel matrices enhances stability, reduces toxicity, and ensures prolonged therapeutic action. This multimodal approach supports the design of advanced antimicrobial materials, suitable for wound dressings, tissue engineering scaffolds, and infection-control applications in clinical settings [99].

### 5.3. MNHHs Against Viruses, Fungi, and Other Microorganisms

Table 6 reports various studies conducted over the past decade have evaluated the effect of *MNHHs* against a broad range of fungal and viral pathogens and other microorganisms such as mycobacteria or parasites. These investigations highlight how physicochemical factors such as NPs’ composition, size, dispersion uniformity, and hydrogel matrix interactions significantly influence antimicrobial outcomes. Table 6 provides a comparative overview of representative formulations, including Ag, Au, ZnO, and Cu NPs, detailing their performance parameters such as MIC values, inhibition percentages, exposure times, and primary mechanisms of action.

As shown in Table 6, MNHHs demonstrate remarkable broad-spectrum antipathogen efficacy, encompassing fungi and viruses. In the case of fungal pathogens, AgNP- and ZnO-based hydrogels show potent antifungal effects, particularly against *Candida* spp. and *Aspergillus niger*. MIC values ranging from 37.5 to 62.5 μg/mL yield strong inhibition responses, with inhibition zones up to 25 mm and effective antimicrobial performance observed within 24–48 h [36,101,102]. These hydrogels promote cell wall degradation, ROS-mediated mitochondrial damage, and ion release, which compromise fungal viability. Notably, polyacrylamide–chitosan hydrogels incorporating AgNPs achieve strong growth suppression of *C. albicans* at 37.5 μg/mL, underscoring the strong correlation between NP dispersion uniformity and antifungal potency [36].

The evaluation of the antifungal activity of hydrogels reinforced with MNPs against fungi and yeasts, particularly species of the genus Candida, is based on standardized methodologies widely accepted in clinical microbiology, such as the Clinical and Laboratory Standards Institute protocols. In this context, the most used methods include (i) the M27 standard for yeasts (broth microdilution), (ii) the M38 standard for filamentous fungi, (iii) MIC and fungicide (MFC) assays, and (iv) biofilm formation and inhibition models. These approaches allow for the quantitative evaluation of the efficacy of nanocomposite hydrogels, as well as direct comparisons between systems based on different MNPs and polymer matrices [36,101].

First, broth microdilution assays based on the CLSI M27 protocol are the standard for determining the MIC against yeasts such as Candida. In this regard, recent studies have shown that the incorporation of MNPs into hydrogels significantly reduces MIC values compared to conventional systems [36]. For example, Bhaskaralingam et al. (2026) reported MIC values as low as 37.5 μg/mL against *Candida albicans* in Ag/Fe_2_O_3_/CS-Cl-PAM hybrid hydrogels, accompanied by zones of inhibition up to 25 mm, demonstrating potent antifungal activity [36]. In contrast, systems without metallic reinforcement or with a lower NP loading tend to exhibit higher MIC values, indicating lower efficacy against fungal infections, particularly under complex physiological conditions.

Additionally, agar diffusion assays, although less standardized for fungi than for bacteria, continue to be used as preliminary comparative tools. In this context, Elmehbad et al. (2023) demonstrated that chitosan hydrogels modified with ZnO NPs exhibit significant inhibitory activity not only against bacteria but also against *Candida albicans*, which confirms the broad-spectrum nature of these systems [101]. However, as with bacteria, these assays have limitations associated with the diffusion of the antifungal agent, especially in highly crosslinked matrices, which may underestimate the actual efficacy of the material [99].

In terms of fungicidal activity, assessed by the minimum fungicidal concentration (MFC) or the logarithmic reduction of fungal load, nanocomposite hydrogels show promising results. Although not all studies report explicit MFC values, it has been shown that multifunctional systems with MNPs can induce significant reductions in *Candida* spp., especially when they combine ion release and reactive species generation [36,99]. For example, systems with metal–phenolic NPs have demonstrated antifungal activity associated with ROS production and alteration of cell integrity, mechanisms that are also effective against yeasts [99].

A critical aspect in Candida infections, the formation of biofilms is a key challenge for Candida species, as these biofilms exhibit significantly greater resistance to conventional antifungal treatments. In this context, hydrogels containing MNPs have demonstrated a remarkable ability to inhibit both the initial adhesion and maturation of fungal biofilms [36,106]. For example, Bhaskaralingam et al. (2026) [36] reported significant inhibition of *Candida albicans* in drug-controlled release systems, suggesting that the combination of MNPs and therapeutic agents enhances ECM disruption. Similarly, multifunctional systems described by Sun et al. (2025) have demonstrated efficacy in eliminating complex biofilms through catalytic mechanisms and localized ROS generation [106].

From a mechanistic perspective, the antifungal activity of nanocomposite hydrogels is based on mechanisms similar to those described for bacteria, although with particularities associated with the cellular structure of fungi. First, the generation of ROS induces oxidative stress that affects the cell membrane and internal organelles of *Candida* spp., causing apoptosis or cell necrosis [99]. Second, the release of metal ions such as Ag^+^ or Zn^2+^ interferes with key enzymes and metabolic processes, affecting cellular respiration and biomolecule synthesis [101]. Third, the direct interaction between NPs and the fungal membrane alters its permeability, facilitating the entry of toxic species and the exit of intracellular components [107]. Finally, these combined mechanisms contribute to the inhibition of biofilm formation, a determining factor in the persistence of Candida infections [36].

From a comparative analysis, the data indicate that the most effective systems against fungi and yeasts are those that integrate (i) MNPs with controlled sizes (<50 nm), (ii) polymer matrices with high swelling and diffusion capacity, (iii) controlled-release mechanisms, and (iv) additional functionalities such as sensitivity to stimuli. In particular, hybrid hydrogels that combine multiple MNPs or that incorporate antifungal drugs show superior performance, both in terms of MIC reduction and biofilm inhibition [36,106].

Taken together, the experimental evidence demonstrates that hydrogels reinforced with MNPs exhibit significant antifungal activity against *Candida* spp., with low MIC values, effective biofilm inhibition, and multifactorial mechanisms of action. These systems represent a promising alternative to conventional antifungal treatments, especially in the context of resistant and biomaterial-associated infections, where the ability to act simultaneously on multiple cellular targets is critical to improve therapeutic efficacy [36,99,101].

In the case of viruses, experimental evaluation is based on standardized methodologies such as (i) plaque assays to quantify plaque-forming units (PFUs); (ii) cytopathogenicity assays (CPEs) to assess infection-induced cell damage; and iii) RT-qPCR for the precise quantification of viral load. These approaches allow for robust characterization of antiviral activity and facilitate comparisons between systems based on free NPs and hydrogel matrices [103,104,108].

From a comparative perspective, experimental results show that antiviral efficacy depends strongly on the structure of the nanocomposite system. For example, ZnO NP-loaded polymeric hydrogels demonstrated viral inhibition close to 100% against HSV-1 and BCoV, compared to approximately 40% in the hydrogel matrix without NPs, while also increasing swelling from 1044% to 1253% and stability up to 96 h [103]. However, more complex systems such as TA-crosslinked GO/ZnO/chitosan nanocomposite hydrogels achieved antiviral inhibition of approximately 86% against HSV-1 with low cytotoxicity, which corresponds to strong antiviral performance and highlights the contribution of synergistic interactions between components [104]. Studies in fish viruses have also shown viral load reductions of between 5 and 330 times at non-cytotoxic concentrations (~25 ng/mL), demonstrating the high sensitivity of these systems to dose and application method [108]. Taken together, these results demonstrate that incorporation into matrices or hybrid platforms improves colloidal stability, prevents aggregation, and enhances antiviral activity.

In the specific context of hydrogels, systems loaded with MNPs show additional advantages associated with controlled release and prolonged interaction with the pathogen. For example, polymeric hydrogels with ZnO NPs have demonstrated viral inhibition close to 100%, compared to approximately 40% in the matrix without NPs, evidencing a clear synergistic effect between the polymer network and the metallic component [103]. Furthermore, the incorporation of ZnO increased the swelling of the hydrogel (from 1044% to 1253%) and its stability (up to 96 h), promoting sustained release and greater interaction with viral particles [103]. This behavior is consistent with the principles described in advanced delivery systems, where the hydrogel matrix regulates bioavailability and enhances therapeutic efficacy [22,81].

At a mechanistic level, the antiviral activity of these systems is based on a multifactorial action: (i) direct interaction of the NPs with viral envelope proteins, blocking adsorption and cellular entry; (ii) generation of ROS, which induces oxidative damage to structural components; and (iii) release of metal ions (Ag^+^, Zn^2+^), which interfere with viral replication and metabolic processes. In hybrid systems such as TA-crosslinked GO/ZnO/chitosan hydrogels, synergistic effects, including ROS generation, viral glycoprotein interaction, and inhibition of viral entry and replication, have been demonstrated, reducing viral internalization and limiting its spread [104,108]. The integration of these mechanisms into hydrogels allows for controlled and sustained release, optimizing efficacy and reducing cytotoxic effects [27,109].

In the case of mycobacteria, particularly *Mycobacterium tuberculosis*, studies are more limited but highly relevant due to the intrinsic resistance of this pathogen. AgNPs supported on mesoporous matrices have been shown to exhibit significant antimycobacterial activity, with lower MIC values in structured systems compared to free NPs [82]. In particular, mesoporous silica-based systems with AgBr showed greater efficacy than core–shell configurations, demonstrating that the distribution of the metal within the matrix directly influences biological activity [110]. This behavior suggests that incorporation into hydrogels could further enhance efficacy through sustained release and longer contact time with the mycobacterial cell [109].

In contrast, evidence regarding activity against parasites remains scarce and represents a significant gap in the literature. Some studies have shown that Ag.MA.CS/PUF nanocomposites can significantly reduce the viability of Leishmania major, achieving approximately 80% reduction in parasite load and a 28% decrease in lesion size in vivo, through ROS mechanisms, membrane damage, and metabolic interference; however, these results have been obtained mainly in structured nanocomposites rather than classical hydrogels [105]. This limitation prevents direct comparisons and highlights the need to investigate specific hydrogel platforms for antiparasitic applications. Finally, in highly resistant microorganisms such as spores, the available information is even more limited. However, the proposed mechanisms mainly ROS generation and metal ion release, are consistent with those observed in bacteria and viruses, suggesting a potential application that has not yet been sufficiently explored experimentally [72,73].

From a critical perspective, the data show that efficacy against unconventional microorganisms is strongly influenced by (i) NP size (greater activity in ranges < 10 nm), (ii) hydrogel structure (swelling and diffusion capacity), and (iii) the presence of controlled-release or stimulus-responsive mechanisms. However, heterogeneity in experimental methods, especially in antiviral and antiparasitic assays, limits comparability between studies and hinders the standardization of results. Overall, although hydrogels with MNPs show high potential against viruses and mycobacteria, significant gaps remain regarding parasites and spores, which constitutes a priority area for future research in advanced antimicrobial biomaterials [103,104,108].

## 6. Biomedical Applications of MNHHs

MNHHs have emerged as multifunctional biomaterials with broad biomedical applications due to the synergistic combination of the physicochemical properties of hydrogels and the unique biological activity of MNPs. Beyond their antimicrobial effects, MNHHs exhibit promising performance in wound healing, tissue engineering, controlled drug delivery, cancer therapy, and regenerative medicine [24,30]. The principal biomedical applications of MNHHs and their therapeutic advantages are summarized in Table 7. The biomedical relevance of MNHHs lies in their ability to integrate multiple therapeutic functions within a single platform. By combining hydrated polymeric networks with bioactive MNPs, these systems can simultaneously prevent infection, support tissue repair, deliver therapeutic agents, and enable disease-targeted therapies, thereby expanding their applicability across diverse areas of regenerative and translational medicine [15,31]. Among the different biomedical applications, wound healing represents one of the most extensively studied fields for MNHHs. Chronic wounds, diabetic ulcers, burns, and infected lesions require biomaterials capable of maintaining a moist environment, preventing microbial colonization, promoting tissue regeneration, and accelerating healing processes. MNHHs fulfill these requirements through the combined action of hydrogels and bioactive NPs [29,61].

In wound management, MNHHs act as multifunctional dressings capable of combining antimicrobial protection with tissue regenerative effects. Their therapeutic performance is enhanced by the incorporation of MNPs, which actively modulate biological processes involved in healing while reducing the risk of infection. In addition, MNPs such as AgNPs, CuNPs, ZnO NPs, and AuNPs contribute broad-spectrum antimicrobial activity, reducing bacterial contamination and biofilm formation at the wound site [50,63,64,97]. For example, AgNP-loaded chitosan hydrogels demonstrated more than 90% inhibition of *S. aureus* and *Escherichia coli*, while simultaneously maintaining favorable biocompatibility and supporting tissue repair [111]. Furthermore, AgNP-containing PVA hydrogels have demonstrated enhanced wound closure, improved tissue regeneration, and favorable histological outcomes, supporting their potential for wound healing applications [61]. The release of Zn^2+^ ions from ZnO NP-integrated hydrogels has been associated with enhanced stem cell proliferation, reduced inflammatory responses, accelerated cell migration, and improved tissue regeneration [64]. Similarly, ZnO NP-containing hydrogels have demonstrated anti-inflammatory effects and promoted cell proliferation and tissue repair processes, supporting their application in regenerative medicine [86]. Additionally, self-healing systems based on oxidized dextran, carboxymethyl chitosan, and AgNP-decorated nanostructures exhibit adhesive and self-repairing properties, thereby increasing residence time at the wound site and reducing the need for frequent dressing replacement [35,88].

MNHHs have also gained attention as controlled drug delivery systems. Beyond antimicrobial applications, MNHHs have emerged as versatile therapeutic carriers capable of improving the local bioavailability of drugs and reducing systemic exposure. Their multifunctional design enables the integration of therapeutic molecules and MNPs within a single platform for localized treatment. The incorporation of MNPs into hydrogel matrices enables sustained and localized release of therapeutic molecules while improving structural stability and encapsulation efficiency. Stimuli-responsive hydrogels capable of responding to pH, temperature, magnetic fields, or near-infrared irradiation have been developed to achieve on-demand release of therapeutic agents [92]. For instance, multilayer hydrogels containing AgNPs demonstrated pH-dependent release profiles that improved release precision and minimized burst effects [89]. Likewise, PEG/PVP hydrogels incorporating TiNPs and polydeoxyribonucleotide (PDRN) exhibited a biphasic release profile characterized by an initial burst followed by sustained release over 96 h, supporting applications in localized tissue regeneration [24].

In tissue engineering and regenerative medicine, MNHHs serve as biomimetic scaffolds capable of emulating the ECM, thereby supporting cellular adhesion, proliferation, and differentiation [29]. In regenerative medicine, MNHHs are increasingly explored as bioactive scaffolds that not only support tissue reconstruction but also provide biological signals that promote cellular responses associated with tissue repair and functional recovery. The incorporation of MNPs improves scaffold performance by enhancing mechanical strength, modulating porosity and swelling behavior, and promoting cell migration and tissue regeneration [34]. For instance, conductive hydrogels containing Ag@rGO nanocomposites have demonstrated electrical conductivity, injectability, self-healing behavior, tissue adhesion, antibacterial activity, and biocompatibility, highlighting their potential for advanced wound dressings and regenerative applications [35]. Moreover, injectable and self-healing MNHHs facilitate minimally invasive administration and adaptation to irregular tissue defects, improving scaffold integration and tissue remodeling [88].

Among the emerging biomedical applications of MNHHs, cancer therapy has attracted considerable attention because these systems enable the combination of localized treatment, controlled therapeutic delivery, and NP-mediated antitumor activity within a single platform. MNPs such as AgNPs, AuNPs, CuNPs, and SeNPs can induce selective cytotoxicity against tumor cells through ROS generation, photothermal conversion, and controlled ion release [31,49]. Thermosensitive hydrogels loaded with AgNPs and ZnO NPs demonstrated significant cytotoxicity against breast cancer cell lines (MCF-7 and MDA-MB-231), reducing cell viability to approximately 37–38% and inducing apoptosis rates of up to 87% [31]. Similarly, injectable nano-hydrogel composites incorporating copper selenide NPs (CuSe NPs) and biomimetic drug-loaded NPs effectively suppressed tumor growth in H22 tumor-bearing mice while exhibiting minimal systemic toxicity, favorable biodistribution, and enhanced photothermal therapeutic performance [49]. These multifunctional systems combine therapeutic delivery with diagnostic or stimuli-responsive functions, expanding the theranostic potential of MNHHs for advanced biomedical applications.

## 7. Toxicity and Biosafety

The toxicity and biosafety of MNHH-based hydrogels are critical considerations for biomedical applications, since interactions between NPs and biological systems may result in both beneficial therapeutic outcomes and undesirable adverse effects. In vitro studies have shown that cytotoxicity depends on factors such as the type of NPs, their concentration, size, and the hydrogel’s ability to modulate their release [31]. For example, thermosensitive systems incorporating AgNPs and zinc oxide have shown a marked reduction in cell viability in breast cancer tumor lines (MCF-7 and MDA-MB-231), reaching values close to 37–38%, with mean inhibitory concentrations (IC_50_) of 14.87 μg/mL for AgNPs and around 19 μg/mL for ZnO, along with apoptosis rates close to 87%, which demonstrates a significant cytotoxic effect associated with mechanisms such as oxidative stress and activation of apoptotic pathways [31]. However, in applications aimed at tissue regeneration or controlled drug release, low cytotoxicity profiles have been reported when NPs are adequately stabilized within the hydrogel matrix. In this regard, PEGDA/gelatin hydrogels with AgNPs showed a negligible influence on cell viability under optimal conditions, attributed to the controlled release of Ag ions (0.35 ppm over 7 days), which limits direct cell exposure and reduces toxic potential [32]. Similarly, hydrogels based on carboxymethylcellulose, acrylamide, and AuNPs exhibited high antibacterial activity (>98%) without showing toxicity to mammalian cells, highlighting the role of the polymer matrix in modulating the cell-nanomaterial interaction [96].

Regarding toxicity mechanisms, several studies agree that the generation of ROS is one of the main factors responsible for cell damage, inducing oxidative stress, DNA alterations, and potential genotoxicity. For example, hydrogels with CuNPs demonstrated increased ROS production as part of their antibacterial mechanism, which, although beneficial against microorganisms, can generate adverse effects in host cells if not properly controlled [63]. In vivo, available evidence suggests that hydrogels with MNPs can exhibit adequate biosafety when designed with controlled release and optimized biocompatibility. Studies in animal models have shown that hydrogels with CuNPs or AgNPs can accelerate wound healing, promote tissue regeneration, and reduce infections without inducing severe inflammation or significant tissue damage [61,97]. Furthermore, advanced systems such as hydrogels with CuNPs and SeNPs have demonstrated effective suppression of tumor growth in murine models, with minimal systemic toxicity and adequate biodistribution, indicating a favorable safety profile for oncology applications [49].

Another relevant aspect is the bioaccumulation and potential prolonged release of metal ions. In this context, hydrogel design plays a fundamental role, since highly cross-linked matrices or those with high encapsulation capacity (>90% in some systems with TiNPs) limit NP migration and reduce their accumulation in non-target organs [24]. Furthermore, approaches including the employment of alternative initiators or the elimination of residual monomers have been found to enhance cytocompatibility and inhibit inflammatory responses, as evidenced in polyacrylamide hydrogels containing liquid MNPs or MoS_2_ that did not show any cyto-toxicity, inflammation, or reactions [95].

On the other hand, results of in vitro and in vivo studies show that most hydrogels containing MNPs show good biocompatibility when parameters like concentration, particle size, and release kinetics are controlled. Cell viability assays (>70%), low hemolysis (<2%), and adequate tissue response support their potential for clinical applications, especially in medical devices, drug delivery, and tissue regeneration [30]. Overall, these results show that MNPs, while generating cytotoxic effects mainly related to ROS and ion release, can be modulated by their incorporation in hydrogel matrices, balancing therapeutic efficacy and biological safety.

## 8. Conclusions and Prospects

MNHHs have become established as advanced multifunctional platforms capable of integrating structural support, antimicrobial activity, and controlled release of therapeutic agents in a single system. The evidence considered suggests that the behavior of these systems is governed by the interplay between the polymer network structure, the nature of the NPs, and the release kinetics and is not influenced by the essential properties of the hydrogel. From an antimicrobial standpoint, MNHHs exhibit high and reproducible efficacies, with inhibition until 99.99% against Gram-positive and Gram-negative bacteria, including clinically relevant pathogens such as *E. coli*, *S. aureus*, and *P. aeruginosa*. In hybrid systems based on AgNPs, inhibition zones of up to 25 mm and MIC values close to 37.5 µg/mL have been reported, while platforms combined with metal oxides can reduce the MIC to as low as 0.78 µg/mL. Moreover, a few systems exhibit long-lasting antimicrobial effects, maintaining inhibition zones of around 13 mm after 60 days, indicating a prolonged release and stability over time. Nevertheless, a comparison shows that the efficiency is not only a function of the metallic load but also of its distribution, the particle size (5–40 nm), and the interaction with the hydrogel matrix.

In physicochemical terms, the incorporation of MNHHs induces significant improvements in the structural properties of the hydrogel, where tensile strength increases. Furthermore, porosity can be adjusted within the range of ~30–100 µm, while swelling capacity varies between 50% and 300% depending on the medium, directly impacting the diffusion of biomolecules. Nevertheless, these mechanical properties do not necessarily result in the same magnitude of enhancement in biological activity, suggesting a partial decoupling between the structural and therapeutic properties. The MNHHs have shown great success in biomedical applications, in particular wound healing, but critical limitations to clinical translation remain, despite these advances. These include potential cytotoxicity associated with the release of metal ions, a lack of standardization in experimental parameters (e.g., antimicrobial and mechanical evaluation conditions), and limited information on biodistribution, bioaccumulation, and long-term effects. In addition, the large-scale reproducibility and the fulfillment of the regulatory standards are still challenging, especially in the cases of systems with intricate architectures or multicomponent production.

In this context, prospects are geared towards the development of smart hydrogels that respond to stimuli, optimized hybrid systems, and sustainable synthesis strategies that reduce the use of chemical reagents and improve biocompatibility. Furthermore, rational design based on structure-property-function correlations will be essential to improve reproducibility and facilitate clinical validation. Taken together, MNHHs represent one of the most promising strategies for developing advanced biomaterials to combat AMR. However, their clinical implementation will depend on the ability to integrate release control, biosafety, scalability, and sustainability into reproducible and standardized systems.

## Figures and Tables

**Table 3:** Advantages and limitations of NPs incorporation methods on hydrogels.

Method of Incorporation	Hydrogel/ NPs	Characteristics Main	Properties Featured	Advantages and Limitations	Ref.
In situ	rGO hydrogel/Ag NPs	Biohybridization with Ag NPs; ecological synthesis	High conductivity, antioxidant activity, and improved catalytic and electrocatalytic efficiency	Advantages: Good NP dispersion and no aggregation; Limitations: Requires precise control of biogenesis and rGO to maintain properties.	[14]
Ex situ	ODex/CMCS/Ag@rGO	Schiff reaction; conductive and self-healing hydrogel	Tissue adhesion, injectable, antibacterial, biocompatible	Advantages: High multifunctionality, good NP integration; Limitations: More complex synthesis, critical control of mechanical properties.	[35]
In situ	PVA/Na-alginate/gelatin/AgNPs	Green synthesis mediated by plant extract	Average size 28 nm, antibacterial, accelerated in vivo healing	Advantages: High biocompatibility and therapeutic efficacy; Limitations: NP size control and reproducibility depend on natural extracts.	[68]
In situ	Pectin-silk fibroin/ ZnO@GA NPs	HRP/H_2_O_2_ enzymatic crosslinking; rapid gelation (<1 min); metal-phenolic interaction	Injectable, self-healing, antibacterial (*S. aureus*), sustained release of Zn^2+^ and gallic acid, mechanical improvement	Advantages: Rapid gelation, uniform NP integration, high biocompatibility. Limitations: Limited scalability, NP size control depends on gelation kinetics.	[69]
In situ	PVA/glycerin/ Cu NPs	Cu^2+^ adsorption and reversible generation of NPs	Flexible, full ion saturation, reversible color change, fluorescent detection	Advantages: Reversible and reusable, technological applications; Limitations: Sensitive to external chemical conditions, limited long-term stability.	[70]
Ex situ	Alginate/CuNi carbon-coated NPs	Loading of preformed NPs	Cytocompatible, antibacterial, accelerates wound healing	Advantages: High antibacterial activity, controlled NP loading; Limitations: NP uniformity in gel may vary, limited-scale production.	[71]
Ex situ	DES catalyzed/NPs	Rapid synthesis of multifunctional hydrogels	High extensibility (4187%), conductivity 21.5 mS/m, self-healing, biodegradable (<62 h)	Advantages: Fast preparation, multifunctional; Limitations: Dependence on eutectic solvents, optimization of mechanical properties required.	[72]
Ex situ	GR@TA-AgNPs@MIH	Room-temperature free radical polymerization; molecular imprinting	Self-adhesion, conductivity, electrochemical detection of methyl parathion with LOD 0.099 μM	Advantages: Specifically functionalized for detection; Limitations: Complexity of synthesis and dependence on dual NPs for functionality.	[73]
Ex situ	Cross-linked chitosan/CuAu MNPs	Adsorption and reduction of metal ions; particles 2.6–4.4 nm	plasmonic catalyst, high selectivity, and recyclability	Advantages: Precise control of NP size, high reproducibility; Limitations: Less uniform NP distribution in the matrix, possible aggregation.	[74]
Ex situ	PEGDA Acrylate/AuNPs	AuNP dimers	Ultra-high SERS sensitivity, portable pesticide detection	Advantages: High sensitivity and applicable to surfaces; Limitations: May require UV initiators and polymerization control.	[75]

Ag@rGO: Silver nanoparticles on reduced graphene oxide; AgNPs: Silver nanoparticles; AuNPs: Gold nanoparticles; CMCS: Carboxymethyl chitosan; CuAu-NPs: Copper–gold nanoparticles; CuNPs: Copper nanoparticles; GA: Gallic acid; ODex: Oxidized dextran; PEGDA: Polyethylene glycol diacrylate; PVA: Polyvinyl alcohol; ZnO@GA NPs: Zinc oxide nanoparticles functionalized with gallic acid; GR@TA-AgNPs@MIH: graphene@tannic acid–silver nanoparticles@molecularly imprinted hydrogel.

**Table 4:** Structural properties and release behavior of reinforced hydrogels.

Hydrogel	Mechanical Properties	Thermal Stability	Properties of NPs on the Hydrogel Structure	Controlled Release Behavior	Relevant Outcome	Ref.
DAT + ZnONPs	Compressive strength 150–200 kPa	Temperature-sensitive	ZnONPs reinforce the hydrogel network through ion-mediated interactions, improving compressive resistance and structural stability	Sustained release Zn^2+^ (≈0.25% *w*/*v*). Sustained Zn^2+^ release regulated by the porous tendon-derived matrix and hydrogel crosslinking density	Zn^2+^ release promotes cell proliferation, tenogenic differentiation, and inflammatory resolution	[86]
OHA-CMCS/AgCD + Met	Adhesive, self-healing	Stable at 25–37 °C	AgCD NPs improve hydrogel adhesiveness, self-healing capacity, and network integration	Dual release: Ag^+^ (~80% in 48 h), Met (~65% in 48 h). Hydrogel matrix enables simultaneous and controlled release of Ag^+^ ions and metformin	Multifunctional hydrogel accelerates healing and offers antibacterial control	[88]
Multilayer hydrogel CS + AgNPs	Laminated architecture maintains integrity	Stable pH 1–7	Multilayer structure improves NP retention and prevents premature structural collapse	Layer-by-layer architecture allows pH-responsive and stepwise Ag^+^ release	Multilayer structure allows controlled and antibacterial release	[89]
CPH + FA-AgNPs	Bioactive; withstands handling	Stable at 25–37 °C	AgNPs improve matrix cohesion and maintain hydrogel integrity during manipulation and application	Sustained release Ag^+^ NP (~97% in 11 days). Controlled diffusion of Ag^+^ through the citrus pectin network enables prolonged antimicrobial activity	Hydrogel promotes healing and optimal biocompatibility	[91]
CS-g-PSBMA + AgNPs Microspheres	Good mechanical integrity; controlled swelling	Temperature-sensitive 25–60 °C	AgNP microspheres act as secondary crosslinking points, improving swelling control and structural uniformity	Ag^+^ release 0.015% in 14 days; Temperature-responsive matrix modulates Ag^+^ diffusion and enables adjustable release profiles	Hybrid design ensures sustained bactericidal activity and anti-biofouling properties	[92]
CS/GO/AgNPs	Maintains shape; dynamic swelling	Stable 25–50 °C	GO and AgNPs reinforce the porous structure, improving swelling dynamics and mechanical resistance	Ag^+^ release 87.4 ppb, rate 0.07%. Porous interconnected network regulates water diffusion and sustained Ag^+^ release	Porous network optimizes water-hydrogel interaction, disinfection, and controlled release	[93]

AgNPs: Silver nanoparticles; ZnONPs: Zinc oxide nanoparticles; DAT: Decellularized Achilles Tendon; CS: Chitosan; CMCS: Carboxymethyl chitosan; OHA: Oxidized hyaluronic acid; AgCD: Silver-doped carbon dots; GO: Graphene oxide; CPH: Citrus pectin hydrogel; FA-AgNPs: Silver nanoparticles synthesized from Fructus Aurantii extract; CS-g PSBMA: Chitosan-graft-poly(sulfobetaine methacrylate); Met: Metformin.

**Table 5:** Antimicrobial Activity of MNHHs.

NP/ Hydrogel	Classification	Target Pathogen	Concentration/MIC/Inhibition (%)	Mechanism Observed	Relevant Outcome	Ref.
AgNP/Thermosensitive hydrogel	Bacteria	*S. aureus*, *E. coli*	MIC = 25 μg/mL; Inhibition ≈ 95%	ROS generation; membrane disruption	Accelerated wound healing, reduced bacterial load	[12]
AgNPs/Hydrogel coating (catheters)	Bacteria	*E. coli*, *K. pneumoniae*, *P. aeruginosa*	>99.99% inhibition; ZOI ≈ 16 mm	Ag^+^ release; antibiofilm	Long-term antibacterial activity	[30]
Ag/Fe_2_O_3_ NPs/CS-cl-PAM hydrogel	Bacteria	*S. aureus*, *B. subtilis*	MIC = 37.5 µg/mL; ZOI ≤ 25 mm	ROS; membrane damage	Higher activity vs. individual NPs	[36]
Fe_2_O_3_ NPs/CH–MAA hydrogel	Bacteria	*A. baumannii*	MIC = 0.78–1.25 µg/mL	ROS; enhanced drug delivery	Strong activity vs. resistant strains	[39]
CuNPs/SG–SA hydrogel	Bacteria	*E. coli*, *S. aureus*	10^6^ CFU/mL reduction	ROS; photothermal + ion release	Effective infection control	[63]
AgNPs/PVA–alginate–gelatin hydrogel	Bacteria	*S. aureus*, *P. aeruginosa*, *K. pneumoniae*, *E. coli*	ZOI = 9–19 mm	Membrane disruption; Ag^+^ release	Accelerated wound healing	[68]
Cu–Ni NPs/Alginate hydrogel	Bacteria	*P. aeruginosa*, MRSA	30–50 mg/mL	ROS generation; ion release	Strong antibacterial activity	[71]
AgNPs/CS-g-PSBMA hydrogel	Bacteria	*E. coli*	Ag loading = 122.6 mg/g; release 33–52 µg/L	Controlled Ag^+^ release	Sustained antibacterial effect	[89]
GO–Ag/Alginate hydrogel	Bacteria	Gram (+)/Gram (−)	ZOI ≤ 39 mm	Membrane disruption; ROS	High antibacterial efficacy	[98]
Co-phenolic NPs/HA hydrogel	Bacteria	*S. aureus*, *P. aeruginosa*	4-log (*S. aureus*); 2-log (*P. aeruginosa*) reduction	ROS; biofilm inhibition	Effective chronic wound treatment	[99]
ZnONPs/ Chitosan hydrogel	Bacteria	*P. aeruginosa*, *S. epidermidis*	MIC ≈ 1.95 µg/mL	ROS; cell wall disruption	Enhanced antimicrobial activity	[101]

AgNPs: Silver nanoparticles; Fe_2_O_3_ NPs: Iron oxide nanoparticles; CuNPs: Copper nanoparticles; Cu–NiNPs: Copper–nickel nanoparticles; ZnONPs: Zinc oxide nanoparticles; GO–Ag: Graphene oxide–silver nanoparticles; Co-phenolic NPs: Cobalt–phenolic nanoparticles; CS: Chitosan; CS-cl-PAM: Chitosan crosslinked polyacrylamide; CS-g-PSBMA: Chitosan-grafted poly(sulfobetaine methacrylate); SG–SA: Sesbania gum–sodium alginate; CH–MAA: Chitosan–methacrylic acid; HA: Hyaluronic acid; PVA: Poly(vinyl alcohol); MIC: Minimum inhibitory concentration; ZOI: Zone of inhibition; ROS: Reactive oxygen species; CFU: Colony-forming units; *E. coli*: *Escherichia coli*; *S. aureus*: *Staphylococcus aureus*; *P. aeruginosa*: *Pseudomonas aeruginosa*; *K. pneumoniae*: *Klebsiella pneumoniae*; *S. epidermidis*: *Staphylococcus epidermidis*; *B. subtilis*: *Bacillus subtilis*; *A. baumannii*: *Acinetobacter baumannii*; MRSA: Methicillin-resistant *Staphylococcus aureus*.

**Table 6:** Comparative efficacy of MNHHs against viruses, fungi, and other microorganisms.

NP/ Hydrogel	Classification	Target Pathogen	Concentration/MIC/Inhibition (%)	Mechanism Observed	Relevant Outcome	Ref.
Ag/Fe_2_O_3_ NPs/Chitosan-cl-polyacrylamide hydrogel	Fungi	*Candida albicans*	MIC = 37.5 µg/mL; inhibition zone ≤ 25 mm	ROS generation; membrane disruption	Enhanced antifungal activity compared to individual NPs	[36]
ZnO NPs/Chitosan-based hydrogel	Fungi	*Candida albicans*	MIC ≈ 1.95 µg/mL	ROS generation; cell wall disruption	Enhanced antifungal and antibiofilm activity; good biocompatibility	[101]
β-AgVO_3_ + AgNPs/Gel	Fungi	*Candida albicans*	MIC = 62.5 µg/mL; antifungal effect at 20× MIC (≈1250 µg/mL)	Ag^+^ ion release; membrane damage	Significant growth inhibition at ≥20× MIC; comparable to 0.12% chlorhexidine	[102]
ZnO NPs/Polymeric hydrogel	Virus	HSV-1, BCoV	Viral inhibition ≈ 100% (HZ); ~40% for control hydrogel	ROS generation; viral binding inhibition; entry blocking	Strong antiviral activity and enhanced hydrogel stability	[103]
TA@ZnO/GO/Chitosan-based hydrogel	Fungi	*Candida albicans*, *Aspergillus niger*	IZ ≈ 21 mm (*C. albicans*); no inhibition for *A. niger*.	Membrane damage; oxidative stress.	Selective antifungal activity (effective against *C. albicans*)	[104]
TA@ZnO/GO/Chitosan-based hydrogel	Virus	HSV-1	Antiviral inhibition ≈ 86%; concentration range 31.25–1000 µg/mL; C_50_ ≈ 216 µg/mL	Binding to viral glycoproteins; entry blockade; inhibition of replication; synergistic effect of TA–ZnO–GO	High antiviral activity with low cytotoxicity	[104]
AgNPs + MA/Chitosan–polyurethane (Ag.MA.CS/PUF) nanocomposite	Protozoa	*Leishmania* *major*	Reduction in lesion size = 28%; reduction in parasitic load ≈ 80%	ROS generation; controlled drug release; synergistic action of AgNPs + MA; parasitic cellular damage	Decrease in amastigotes (from 6+ to 1.16+); improved healing and survival (≈100%).	[105]

AgNPs: Silver nanoparticles; β-AgVO_3_: Beta silver vanadate; BCoV: Bovine coronavirus; CC_50_: Half maximal cytotoxic concentration; CS: Chitosan; GO: Graphene oxide; HSV-1: Herpes simplex virus type 1; IZ: Inhibition zone; MA: Meglumine antimoniate; MIC: Minimum inhibitory concentration; PUF: Polyurethane foam; ROS: Reactive oxygen species; TA: Tannic acid; ZnO: Zinc oxide.

**Table 7:** Representative biomedical applications of MNHHs and their therapeutic advantages.

Biomedical Application	Hydrogel Composition	Main Function of MNPs	Therapeutic Advantages	Representative Outcomes	Ref.
Controlled drug delivery	PEG/PVP hydrogel containing TiNPs and PDRN	Regulation of drug diffusion and hydrogel stabilization	Sustained and localized therapeutic release	Biphasic release profile with sustained PDRN release for up to 96 h	[24]
Anticancer therapy	Thermosensitive hydrogel containing AgNPs and ZnO NPs loaded with withaferin-A	ROS generation and apoptosis induction	Selective cytotoxicity against tumor cells	Cell viability reduced to 37–38% and apoptosis rates approaching 87% in breast cancer cells	[31]
Tissue engineering scaffold	Conductive CMCS/ODex hydrogel containing Ag@rGO nanocomposites	Electrical conductivity, antibacterial activity, and mechanical reinforcement	Injectable, self-healing, adhesive, and biocompatible scaffold for tissue regeneration	Conductive behavior, self-healing properties, and excellent cell compatibility	[35]
Theranostic applications	Injectable nano-hydrogel composite containing CuSe NPs	Photothermal therapy, ROS generation, and targeted drug delivery	Tumor suppression with minimal systemic toxicity	Effective inhibition of tumor growth in H22-bearing mice with favorable biodistribution and biocompatibility	[49]
Wound healing and tissue regeneration	PVA hydrogel incorporating AgNPs	Antibacterial activity and tissue repair support	Enhanced wound closure and tissue regeneration	Improved wound healing rates and histological evidence of tissue regeneration	[61]
Antibacterial wound dressing	SG/SA hydrogel containing CuNPs	Controlled Cu^2+^ release, ROS generation, and photothermal antibacterial activity	Prevention of infection and promotion of wound healing	Effective antibacterial activity against *E. coli* and *S. aureus* with accelerated wound closure	[63]
Regenerative medicine and tendon repair	DAT hydrogel reinforced with ZnO NPs	Zn^2+^-mediated bioactivity and anti-inflammatory effects	Promotion of stem cell proliferation, migration, and tissue regeneration	Enhanced TDSC proliferation, reduced IL-6 and TNF-α expression, and improved tendon repair	[86]
Self-healing wound scaffold	OHA-CMCS/AgCD hydrogel	Antibacterial activity and structural reinforcement	Adhesive and self-repairing properties with prolonged residence time	Sustained Ag^+^ and metformin release, reduced inflammation, and accelerated diabetic wound healing	[88]
Stimuli-responsive drug delivery	CS-g-PSBMA hydrogel containing AgNPs	Temperature-responsive silver release	Adjustable and prolonged antimicrobial activity	Controlled Ag release with sustained antibacterial efficacy and anti-biofouling performance	[92]
Wound healing	Chitosan hydrogel loaded with AgNPs	Antibacterial activity and infection control	Accelerated healing, reduced bacterial colonization, and improved tissue regeneration	>90% inhibition of *S. aureus* and *E. coli* with favorable biocompatibility	[111]

AgCD: Silver-doped carbon quantum dots; Ag@rGO: Silver nanoparticle-decorated reduced graphene oxide; AgNPs: Silver nanoparticles; CMCS: Carboxymethyl chitosan; CS-g-PSBMA: Chitosan-graft-poly(sulfobetaine methacrylate); CuNPs: Copper nanoparticles; CuSe NPs: Copper selenide nanoparticles; DAT: Decellularized Achilles tendon extracellular matrix; ODex: Oxidized dextran; OHA: Oxidized hyaluronic acid; PEG: Poly(ethylene glycol); PDRN: Polydeoxyribonucleotide; PVA: Polyvinyl alcohol; ROS: Reactive oxygen species; SA: sodium alginate; SG: sesbania gum; ZnO NPs: Zinc oxide nanoparticles.

## Data Availability

No new data were created or analyzed in this study. Data sharing is not applicable to this article.
